# Factors associated with mental health among young adults: Cross-country longitudinal evidence from Ethiopia, India, and Peru

**DOI:** 10.1016/j.ssmph.2026.101911

**Published:** 2026-03-24

**Authors:** Marta Favara, Richard Freund, Juliana Quigua, Alan Sánchez

**Affiliations:** a University of Oxford, Queen Elizabeth House. 3 Mansfield Road, Oxford, OX1 3TB, United Kingdom; b University of Cape Town, South Africa; c MIDE Development, United Kingdom; d University of Oxford, United Kingdom; e University of Oxford, United Kingdom; f Grupo de Analisis para el Desarollo (GRADE), Peru

**Keywords:** Mental health, Anxiety, Depression, Early predictors, Low- and middle-income countries

## Abstract

This study investigates contingent and longitudinal predictors of mental health in early adulthood, using unique, harmonized panel data from the Young Lives study in Ethiopia, India, and Peru, spanning over two decades across diverse settings. It accounts for factors and events occurring during the most significant developmental stages, from infancy through childhood and adolescence. It focuses on self-reported symptoms of anxiety and depression, measured amid unprecedented turmoil, during and after the global COVID-19 pandemic and Ethiopia's civil war. High rates of anxiety and depression were sustained over the whole period (2021-2024) in Peru, the country reporting the highest prevalence of mental health issues; they remained stable in India and showed post-conflict deterioration in Ethiopia. Original findings identify key life-stage predictors. In early life, poor caregiver's mental health and higher household wealth are associated with increased adult anxiety, particularly in Peru. During adolescence, strong parent-child relationships and a sense of pride protect against adult anxiety, while academic performance shows weaker associations. In adulthood, neuroticism is a consistent risk factor across all countries, whereas grit is uniquely protective in India. Across all settings, experiences of violence and economic shocks are strong correlates of poor mental health. Significant sex disparities exist in Peru and India, where females report worse outcomes, yet no such gap was found in Ethiopia. These findings emphasize the need for gender-sensitive, context-specific mental health policies in low- and middle-income countries, highlighting the need to address risk factors throughout the life course to promote young people's mental well-being.

## Highlights

• Reported anxiety and depression symptoms vary by country over time: highest in Peru, rising post conflict in Ethiopia, stable in India.
• Gender disparities are substantial, with women reporting significantly worse mental health in Peru and India.
• Early-life conditions affect mental health in adolescence and young adulthood, with key protective and risk factors emerging over time.
• Economic shocks and violence, including IPV and conflict in Ethiopia, are strong risk factors for poor mental health.
• Evidence highlights the need for gender-sensitive, context-specific mental health policies across the life course.

## 1 Introduction

Recent years have seen a substantial rise in awareness and research on global mental health, with mental health difficulties increasingly being recognized as a major public health issue (Patel et al., 2018). Their causes are now widely understood to be multifaceted, involving an intricate interplay of biological, socioeconomic, and psychological factors (Lund et al., 2018).

Among these difficulties, anxiety and depression disorders are particularly significant. Together, they constitute two of the most common mental health conditions globally and rank among the leading causes of disability, affecting roughly 300 million people—around 4% of the world's population.^1^ Addressing these conditions has therefore become a priority for policymakers worldwide. Understanding their determinants and trajectories is crucial for informing prevention and intervention strategies.

The COVID-19 pandemic underscored the urgency of strengthening our understanding of mental health. It exposed stark differences in mental health outcomes across countries, including anxiety and depression, reflecting varying socio-economic, cultural, and policy contexts (Bernardini et al., 2021; Lindert et al., 2021; Nochaiwong et al., 2021). While substantial progress has been made in understanding the predictors of adverse mental health outcomes within individual high-income countries (Cadman et al., 2024; Currie & Morgan, 2020), much less is known about how they differ across countries, particularly in low- and middle-income countries (LMICs). Furthermore, most research on this topic in LMICs has typically used cross-sectional data and focused on a narrow set of factors that may determine mental health (Fryers & Brugha, 2013), such as socio-economic characteristics and gender (Campbell et al., 2021; Das et al., 2009; Patel & Kleinman, 2003).

These studies are unable to account for factors and events occurring during the most significant developmental stages, from infancy through childhood and adolescence. Moreover, cross-country comparisons are further complicated by inconsistencies in survey design, concerns over sample comparability, and the scarcity of harmonized data across diverse settings. We partially address this knowledge gap by utilising rich panel data on a cohort of individuals tracked for over two decades prior to adulthood across three countries: Ethiopia, India, and Peru. These countries provide a rich picture of the developing world, where mental health is likely to become increasingly relevant in the coming decades. Specifically, this paper has two main objectives. First, we analyse descriptively the patterns of at least mild symptoms of anxiety and depression (in the last two weeks), focusing on differences in levels and changes over time across the three countries. Second, we use panel-based decompositions employing detailed child-level data collected over more than two decades, to determine how much mental health differences in early adulthood are associated with individual and household circumstances across childhood, adolescence, and young adulthood.

Our evidence suggests that reported symptoms of anxiety and depression vary significantly across countries and over time, with Peru exhibiting the highest prevalence, Ethiopia showing post-conflict deterioration, and India remaining relatively stable. Early-life conditions such as caregiver mental health and household wealth are associated with later mental health, particularly in Peru. During adolescence, strong parent-child relationships and a sense of pride are correlated with better adult mental health, while academic performance shows weaker associations. In young adulthood, personality traits—especially neuroticism—are consistently associated with worse mental health, while grit shows positive associations in India. Economic shocks (e.g., job loss, food insecurity, crop failure) and experiences of violence, including intimate partner violence and, in Ethiopia, conflict-related violence, emerge as strong correlates. Sex disparities are substantial, with females reporting significantly worse mental health in Peru and India.^2^ While some predictors are consistent across countries, others are highly context-specific, emphasizing the importance of local economic and social conditions. Overall, the findings underscore the need for integrated, sex-sensitive, and context-specific mental health interventions that span the life course, particularly in LMICs with limited mental health infrastructure.

## 2 Background

To contextualize our later analysis of factors associated with mental health in the three countries, we begin by providing an overview of the socio-economic context over the past two decades (Section 2.1), a discussion of the mental health context (Section 2.2), and a description of the most recent challenges in the countries (Section 2.3).

### 2.1 Socio-economic context of the study countries

Over recent decades, Ethiopia, India and Peru have sustained high rates of economic growth, with average annual rates exceeding 4% between 2002 and 2023, as reported by the World Bank Indicators. Following the World Bank classification of GNI per capita in US dollars, both India and Peru are currently classified as middle-income countries. However, India was low-income in the early 2000s, while Peru transitioned from lower-to upper-middle-income status. Despite Ethiopia's rapid GDP growth (an average annual growth rate of 8.5% since 2002), it remains classified as a low-income country.

At the same time, these countries face similar challenges, including high levels of inequality by socioeconomic and ethnic background, low-quality education and employment opportunities, and vulnerability to economic shocks. In contrast, they differ in some important dimensions. India and Ethiopia are predominantly rural, with sizable agricultural sectors, whereas Peru is largely urban. While all three countries are multi-ethnic, differences in child outcomes by caste and sex are particularly distinctive in India.

### 2.2 Mental health awareness and infrastructures in the study countries

Mental health has historically been overlooked in public health discussions, particularly in LMICs, gaining legitimacy as a critical area of concern only in recent decades. The pace of this recognition varies by country due to cultural attitudes, resources, and public health priorities, resulting in ongoing disparities in how mental health is perceived and addressed.

In Ethiopia, mental health remains a relatively underdeveloped field, with limited public discourse and scarce resources (Yitbarek et al., 2021). Although the government implemented the Ethiopian National Mental Health Strategy in 2012, no resources were allocated to its implementation (WHO, 2022a). Additionally, Ethiopia lacks a stand-alone mental health law and psychiatric units in general hospitals. This is reflected in its underdeveloped mental health infrastructure, with only 0.68 mental health professionals per 100,000 people, and only 46 practicing psychologists in the country (0.04 per 100,000 population, compared to an average of 60 mental health workers per 100,000 people in high-income countries (WHO, 2021).

In India, mental illness has long been associated with stigma, ignorance, and superstition (Dhyani et al., 2022). Despite several government-led programs, including the Mental Healthcare Act of 2017, the country still suffers from inadequate mental health infrastructure. With just 0.75 psychiatrists per 100,000 people (as cited in Chakrapani & Bharat, 2023) and 898 clinical psychologists nationwide (MoH&FW, 2017; as cited in Chakrapani & Bharat, 2023), India fares slightly better than Ethiopia in terms of mental health resources. Nevertheless, the current supply is critically low, with substantial gaps in community care services and insufficient support for family caregivers, leading to unmet needs and treatment disparities (Chakrapani & Bharat, 2023).

Peru is the most economically developed of the three study countries, as reflected in its comparatively greater attention to mental health, though many of these developments have occurred recently. The country has both a stand-alone mental health plan and a mental health law, supported by allocated financial and human resources, with a dedicated authority overseeing implementation and publishing annual findings (WHO, 2022b). This commitment is reflected in the markedly higher availability of mental health professionals. Peru has 25 mental health professionals per 100,000 people – more than 36 times that of Ethiopia – and 5221 practicing psychologists, equivalent to 16 per 100,000 population. Overall, Peru has the most developed mental health system among the countries studied by Young Lives, although it still lags behind many high-income countries.

### 2.3 Most recent challenges in the Young Lives study countries

Since the onset of the COVID-19 pandemic, the three study countries have faced markedly different experiences. In 2020, Peru bore the heaviest health burden of the pandemic, recording the world's highest per capita levels of COVID-19 cases and deaths. In comparison, Ethiopia and India managed to contain the spread of infections much more effectively during the same period. As of December 30, 2020, cases per million population in India and Ethiopia stood at 7187 and 984, respectively, compared to 30,139 in Peru. Between January and July 2021, despite early success in controlling the virus in 2020, India experienced a devastating second wave of infections in April and May 2021. By December 2021, cases in India had increased to 24,441 per million.

The pandemic, together with the economic restrictions it triggered, caused major social and economic disruptions. In 2020, prolonged nationwide lockdowns contributed to steep GDP declines of 11.0% in Peru and 6.6% in India, well above the average decline of 1.3% recorded among LMICs. Over the same period, youth unemployment surged by 71% in Peru and 9.6% in India relative to 2019 levels. In 2021, a shift towards more decentralised decision-making and the easing of restrictions supported an economic rebound, with GDP expanding by 13.3% in Peru and 8.9% in India. Despite this, youth labor market outcomes did not follow the same recovery path: youth unemployment in India continued to rise to a record 28.3%. In comparison, in Peru, it remained elevated at 11.2%, above pre-pandemic rates. 55.

While the COVID-19 pandemic was a dominant concern globally, it was overshadowed in Ethiopia by the outbreak of civil war in the Tigray region. In early November 2020, amidst a broader period of ongoing instability, war broke out between the federal government's Ethiopian National Defence Forces (ENDF) and the Tigray People's Liberation Front. Most of the Tigray region was an active war zone and became inaccessible to humanitarian assistance. By mid-2021, the violence had spread to the neighbouring Amhara and Afar regions, compounding social and economic strain. A cessation-of-hostilities agreement was signed in November 2022, ending two years of civil war. However, conflict persists, especially in Amhara, with ongoing hostilities between the ENDF and rebel forces, causing continued suffering (UN General Assembly, 2023).

## 3 Data

### 3.1 Young Lives

Young Lives is a longitudinal study that has collected data on two cohorts of children – born in 1994/95 and 2001/2 – since 2002 in Ethiopia, India (Andhra Pradesh), Peru, and Vietnam.^3^ In this paper, we use data on the 2001/02 cohort from Ethiopia, India, and Peru, known as the ‘Younger Cohort’.^4^ In each country, the initial sample included ∼2000 children aged about 1 year old who were purposively selected to oversample poor families. To establish the sample, 20 clusters (districts in Peru, *mandals* in India, and *weredas* in Ethiopia) were selected in each country and, within each cluster, a random area was chosen. Households in the area were contacted until approximately 100 families with a child aged 6 to 18 months agreed to participate in the study.

Although the samples are not statistically representative at the national level, detailed comparisons with nationally representative surveys — including Demographic and Health Surveys and census data — Young Lives samples are slightly better-off in terms of access to basic services but are often less advantaged in asset ownership, consistent with the study's design. For example, the Young Lives samples generally displayed slightly better access to public services — such as electricity and water in Ethiopia and Peru — than national averages. Conversely, households in Ethiopia were found to own less land and livestock than the national average, and caregivers in India were less likely to have completed primary schooling compared to the national benchmark. Nonetheless, despite these differences, these assessments conclude that the Young Lives samples effectively capture substantial variation in living standards, urban–rural residence, and caregiver education and literacy, akin to the variability found in the general populations across the study countries (for more details, see Escobal & Flores, 2008; Kumra, 2008; Outes-Leon & Sanchez, 2008).

The first survey took place in 2002, immediately after the households were enrolled, with further rounds of in-person data collection in 2006/7, 2009/10, 2013/14, and 2016/17. In all rounds, three main questionnaires were used. The child questionnaire collected information on health, anthropometric measures, and individual attributes. The household questionnaire gathered details on caregivers, household structure, and socioeconomic conditions. Finally, the community questionnaire collected data on infrastructure and demographic, socioeconomic, and environmental features. Survey instruments and field protocols were standardized across all study countries, ensuring comparability in measurement and data quality over time.

Following the COVID-19 outbreak, a five-part phone survey was conducted over 2020/21 (Favara et al., 2021). At the time, individuals were aged between 18 and 19. In 2023/24, Young Lives conducted a seventh survey round, the first in-person round since 2016. This round focused on how the participants were faring in young adulthood. In general, the fieldwork was not affected by external factors. However, the Young Lives team was unable to collect face-to-face data at two sites in the Amhara region of Ethiopia due to ongoing conflict between federal forces and a local militia. Consequently, the team opted to implement a phone survey at these sites, administering a reduced version of the survey round in this case, including, for instance, a full version of the anxiety scale but a reduced version of the depression scale.

Attrition rates across the seven rounds of the Younger Cohort are relatively low over this two-decade period of data collection: 9.2% in India, 17.1% in Peru, and 23.2% in Ethiopia. In Round 7, the primary drivers of attrition were internal and international migration, with most of the “lost” participants having relocated to new addresses, making them difficult to trace further. This is perhaps expected as the Young Lives participants are now young adults who have adopted diverse lifestyles and are increasingly mobile and widely dispersed. Table A.1 (online appendix) presents characteristics of our analytical sample in each country. The sample is mainly urban in Peru and mainly rural in India and Ethiopia. By age 22, the largest proportion of Young Lives participants who completed at least secondary school is observed in Peru, followed by India and then Ethiopia.

### 3.2 Measurement of mental health

Beginning in 2020, anxiety and depression symptoms were assessed using two standardized instruments: the Generalized Anxiety Disorder-7 (GAD-7) for anxiety and the Patient Health Questionnaire-8 (PHQ-8) for depression. The GAD-7 scale captures how often seven anxiety symptoms occurred during the previous two weeks, while the PHQ-8 records the frequency of eight depression symptoms within the same timeframe.^5^ Both scales have been previously validated and used in the three study countries (Carroll et al., 2020; De Man et al., 2021; Mughal et al., 2020; Villarreal-Zegarra et al., 2023, 2024).^6^ Using these instruments, participants can be classified into reporting symptoms that are compatible with “minimal”, “mild”, “moderate”, and “severe” anxiety and/or depression.^7^

In this paper, the primary mental health outcomes (measured in 2023/24) were collected through in-person interviews using Young Lives' standard protocols. Trained fieldworkers administered these instruments face-to-face after confirming respondent privacy and confidentiality. The lagged mental health measures included in the value-added specifications were collected during the 2020 COVID-19 phone survey. Validation analyses conducted on the phone-based implementation in the same cohort reported high internal consistency across all study countries and no evidence of differential item functioning (Porter et al., 2021), providing reassurance that cross-country comparability was retained despite the change in mode of administration. Nonetheless, as with any cross-cultural application of self-reported mental health scales, the possibility of minor mode-related differences in reporting and variation in symptom expression across settings cannot be entirely excluded.

## 4 Prevalence and trends in mental health: 2020-2024

**Fig. 1:**
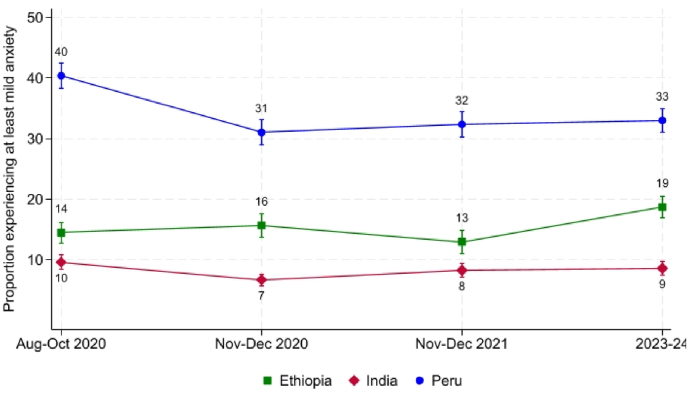
Proportion experiencing at least mild anxiety symptoms, 2020-2024 *Notes*: Analysis is performed on a balanced sample of Young Lives respondents who are present in all four rounds. Vertical bars represent 90% confidence intervals.

**Fig. 2:**
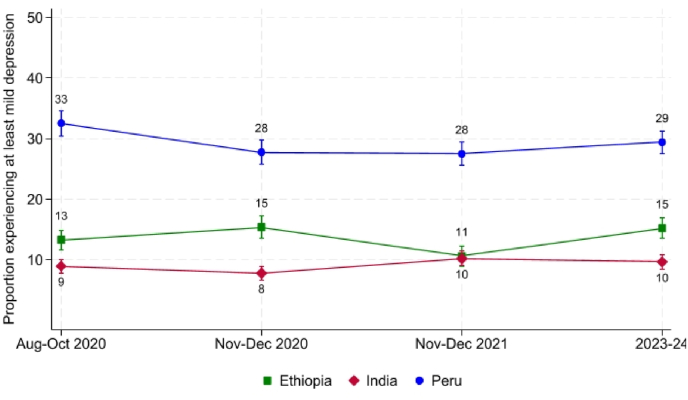
Proportion experiencing at least mild depression symptoms, 2020-2024 *Notes*: Analysis is performed on a balanced sample of Young Lives respondents who are present in all four rounds. Vertical bars represent 90% confidence intervals.

Before delving into the detailed empirical work, we first present descriptive findings on the levels and trends in anxiety and depression across the three study countries. Fig. 1, Fig. 2 present the proportion of participants experiencing symptoms consistent with at least mild anxiety and at least mild depression, respectively.^8^ These binary cut-offs align with clinical diagnostic criteria and have been widely used in recent research using the Young Lives data (Favara et al., 2022; Freund et al., 2025; Porter et al., 2021, 2022). Both figures depict trajectories of mental health between 2020 and 2023. During this period, cohort participants transitioned from ages 19 to 22. Therefore, a main limitation of the analysis is that it cannot separate the effects of events happening between 2020 and 2023 (period effects) from changes that are part of stable developmental paths during young adulthood (age effects) when looking at changes over this time.

During the first wave of data collection amidst the pandemic, in August-October 2020, participants in Peru reported the highest levels of both anxiety and depression by a clear margin. At that time, 40% of the Peruvian sample exhibited symptoms consistent with at least mild anxiety, compared to 14% in Ethiopia and 10% in India. Similarly, 33% of Peruvian respondents reported symptoms compatible with at least mild depression, while Ethiopia and India reported levels comparable to those observed for anxiety.

By November-December 2020, levels of both anxiety and depression had declined slightly in Peru, to 31% and 28%, respectively. Yet, the country continued to stand out with rates well above those in Ethiopia and India. The fact that reported mental health was substantially worse in Peru during late 2020 might be linked to the peak of the COVID-19 pandemic in the country during this period, though there might be other explanations.

At the end of 2021, participants in Peru continued to report the highest prevalence of anxiety and depression, with rates stabilizing at approximately 32% and 28%, respectively. India experienced a marginal increase in both mental health difficulties, possibly also reflecting its heightened exposure to the pandemic in 2021, but levels remained low at around 8%-10%. Lastly, participants in Ethiopia saw a decline in anxiety and depression symptoms, with anxiety falling from around 16% to 13%.^9^ ^10^

By 2023/24, reported levels of mental health in Peru remained consistent with those at the end of 2021, with approximately 33% and 29% of participants reporting symptoms of mild anxiety and depression, respectively. Similarly, mental health in India stabilized, with around 10% of respondents reporting at least mild anxiety and depression. In Ethiopia, however, a marked deterioration in mental health was observed, with the proportion of individuals experiencing mild anxiety and depression rising to 19% and 15%, respectively. One potential explanation is the devastating civil war that plagued the country in recent years—but, as in the other two countries, there might be other explanations, including age-related changes.

Overall, our descriptive findings reveal substantial changes in mental health among participants over the four-year period, which correlate with the evolution of the COVID-19 pandemic in India and Peru, and the civil war in Ethiopia. It remains plausible that the observed cross-country differences may, at least in part, reflect measurement or reporting artefacts. Although the use of standardized survey instruments and a harmonized sampling design mitigates concerns about poor comparability, we cannot entirely rule out cross-cultural differences in how respondents interpret the questions or report symptoms.

**Fig. 3:**
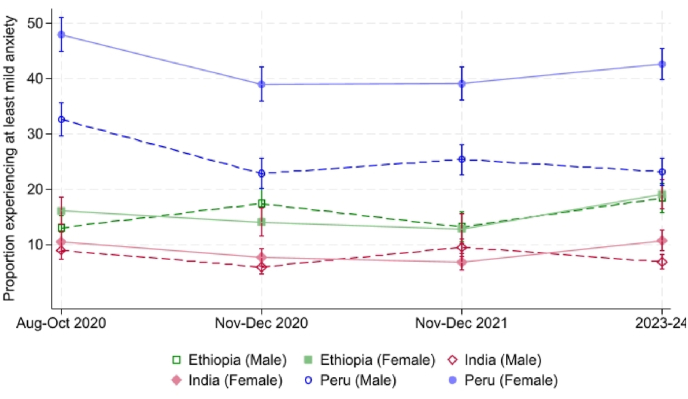
Proportion experiencing at least mild anxiety symptoms, by sex *Notes*: Analysis is performed on a balanced sample of Young Lives respondents who are present in all four rounds. Vertical bars represent 90% confidence intervals.

Fig. 3 depicts the proportion of respondents experiencing at least mild anxiety disaggregated by sex. The figure highlights that, while the prevalence of participants reporting symptoms compatible with at least mild anxiety is generally higher for both sexes in Peru compared to Ethiopia and India, females are markedly worse off in Peru. Indeed, by 2023/24, anxiety levels among Peruvian males are comparable with those observed for both sexes in Ethiopia, while Peruvian females exhibit significantly higher levels, at just over 40%. The role of sex and other factors will be examined in greater detail in the upcoming sections.

## 5 Empirical methodology

Understanding mental health requires accounting for factors measured at different developmental stages, as well as recognizing the dynamic interplay between individual, social, and structural factors. Informed by existing literature—primarily from high-income contexts—we identify three key life stages: early childhood, adolescence, and young adulthood. Each stage encompasses factors that may be associated with mental health in adulthood. Our goal is to trace how experiences at these stages are associated with mental health in early adulthood and to examine variations across the three countries. We also highlight sex as an important factor associated with mental health.

The conceptual framework guiding our variable selection is presented in Appendix A.3. Predictors were grouped according to when they were first measured and when theory and prior evidence suggest they are most relevant for mental health. We acknowledge that some variables may plausibly operate across multiple life stages; however, we assign each to the stage where we believe the expected effect is strongest or best captured in our data. To capture that some predictors evolve over time, and that these trajectories may influence adult mental health, we also include variables summarising changes in key dimensions: the change in household wealth, the change in body mass index (BMI), and an indicator of whether the participant has migrated locations over the study period. These measures allow us to proxy cumulative or transitional experiences that span multiple life stages.

For each country, we estimate the following model via Ordinary Least Squares:

(1) $${MH}_{i}=\beta_{0}+\beta_{1}S_{i}+\beta_{2}{EL}_{i}+\beta_{3}{AD}_{i}+\beta_{4}{YA}_{i\;}+\theta_{e}+\delta_{r}+\mu_{i}\text{,}$$

where the dependent variable, ${MH}_{i}$, is a binary variable taking the value of one if individual *i* reports symptoms compatible with at least mild anxiety (or depression) at age 22. We estimate three specifications introducing three sets of explanatory variables sequentially, always including an indicator of sex ($S_{i}$). First, we introduce ${EL}_{i}$**,** which is a vector of variables corresponding to early-life predictors of anxiety (and depression) at the household or individual level. ${EL}_{i}$ includes maternal (for Peru) or caregiver's (for Ethiopia and India) literacy and mental health, wealth index of the household (Briones, 2017), and the place of residence (urban/rural) - all measured when participants were aged one.

We next include ${AD}_{i}$, a vector of adolescent experiences measured at age 15. This includes an index of subjective perceptions of parent-child relationships, an index of socioemotional competency (pride and agency), school enrolment, mathematics scores, weekly studying hours, and the participant's BMI, expressed in Z-scores. Furthermore, to capture physical changes during adolescence, we include a variable indicating changes in BMI between ages 5 and 15. Lastly, we introduce ${YA}_{i\;}$, which denotes a vector of variables capturing young adulthood characteristics and experiences measured at age 22. ${YA}_{i\;}$ includes interpersonal trust, indices of personality traits (neuroticism and grit), household food insecurity, exposure to shocks experienced in the last seven years (since 2016), hours spent on caregiving or domestic chores, whether the participant has a disability, exposure to different forms of violence—intimate partner violence, online bullying, and exposure to conflict-related violence in Ethiopia —, risky behaviours (consumption of alcohol), whether the participant works, study, both or neither, education attainment, whether the participants sleeps 8 or 9 h, marital status, and fertility. In addition, we measure changes in the wealth index between ages one and 22 and also include an indicator that takes the value of 1 if the participant migrated between ages one and 22, and 0 otherwise.^11^

We also control for caste/ethnicity^12^ by including ethnic fixed effects ($\theta_{e}$), and account for the region of residence ($\delta_{r})$ to control for the influence of geographical location. This is especially important in Ethiopia, where the armed conflict has been concentrated in the northern regions of Tigray and Amhara.

Additionally, we estimate a value-added model for each country, controlling for lagged anxiety (depression) measured in 2020/21. Including the lagged mental health score follows common practice in longitudinal mental health and epidemiological research, where baseline symptom levels are used to capture persistence and pre-existing vulnerability (Ohrnberger et al., 2017). This approach aligns with dynamic health models, in which prior mental health serves as a summary measure of accumulated exposure and individual susceptibility, thereby reducing confounding by time-invariant factors. In this sense, our specification is consistent with the value-added modelling framework widely used in applied microeconometrics (Todd & Wolpin, 2003) but rooted in the epidemiological logic of conditioning on baseline risk. However, we acknowledge that conditioning on prior measures of mental health does not eliminate all sources of confounding — particularly unobserved time-varying factors between waves — so estimates should be interpreted as adjusted associations that move closer to causal interpretations, rather than definitive causal effects.

## 6 Results

### 6.1 Early-life, adolescent, and young adult factors associated with mental health

Our primary findings focus on the partial correlations associated with anxiety (Table 1).^13^ For each country, Column (1) controls for sex and early-life conditions, Column (2) expands the specification to include adolescent factors and experiences, and Column (3) further adds early adulthood experiences. A formal test of the differences in the coefficients across countries observed in Table 1 is reported in the online Appendix (Table A.5). In the text, we refer to these tests only when they help identify meaningful heterogeneity across countries. The analogous set of results for depression are presented in Table A.6 and Table A.7 of the online Appendix. For expositional clarity, these results are discussed at the end of this section, after results for anxiety are presented.^14^

**Table 1:** Factors affecting the likelihood of exhibiting anxiety (at least mild, %).

	Ethiopia			India			Peru		
	(1)	(2)	(3)	(1)	(2)	(3)	(1)	(2)	(3)
Female	0.015	0.021	−0.036	0.038∗∗∗	0.034∗∗	0.038∗∗	0.200∗∗∗	0.188∗∗∗	0.059∗∗
	(0.021)	(0.023)	(0.025)	(0.014)	(0.014)	(0.019)	(0.023)	(0.025)	(0.027)
**Early life conditions (Age 1, Round 1)**									
Caregivers' Mental Health Score (Higher = Worse)	0.002	0.002	0.000	−0.000	−0.001	−0.004∗∗	0.011∗∗∗	0.010∗∗∗	0.005∗
	(0.002)	(0.002)	(0.002)	(0.002)	(0.002)	(0.002)	(0.003)	(0.003)	(0.003)
Caregiver is literate	−0.057∗∗	−0.051∗	−0.035	−0.013	−0.009	−0.020	0.009	0.017	0.011
	(0.027)	(0.027)	(0.026)	(0.016)	(0.017)	(0.017)	(0.032)	(0.032)	(0.030)
Wealth Index	−0.046	0.002	0.110	−0.046	−0.029	0.020	0.170∗∗∗	0.188∗∗∗	0.219∗∗
	(0.098)	(0.103)	(0.121)	(0.051)	(0.053)	(0.084)	(0.063)	(0.065)	(0.094)
Urban	0.113∗∗∗	0.118∗∗∗	0.095∗∗	0.011	0.005	−0.019	0.030	0.024	0.075∗∗
	(0.038)	(0.038)	(0.040)	(0.021)	(0.021)	(0.022)	(0.034)	(0.034)	(0.038)
**Adolescence factors and experiences (Age 15, Round 5)**									
Parent-child relationship		0.007	0.021		−0.029∗∗	−0.012		−0.050∗∗	−0.031∗
		(0.020)	(0.020)		(0.011)	(0.011)		(0.020)	(0.018)
Pride Index (z-score)		−0.051∗∗∗	−0.034∗		0.003	−0.004		−0.024	−0.006
		(0.020)	(0.019)		(0.011)	(0.010)		(0.022)	(0.019)
Agency Index (z-score)		0.037∗	0.030		0.004	−0.002		0.032	0.027
		(0.021)	(0.020)		(0.013)	(0.013)		(0.024)	(0.021)
Math Test (% Correct)		0.001	0.001		−0.001∗∗∗	−0.000		−0.001	0.000
		(0.001)	(0.001)		(0.000)	(0.000)		(0.001)	(0.001)
Study Hours (Last Week)		−0.022∗∗∗	−0.017∗∗		0.002	0.003		−0.005	−0.004
		(0.008)	(0.007)		(0.004)	(0.004)		(0.006)	(0.006)
Enrolled in School		0.103	0.070		0.031	0.029		−0.054	−0.090
		(0.065)	(0.061)		(0.047)	(0.048)		(0.089)	(0.080)
BMI (z-score)		−0.002	0.004		0.006	0.004		0.002	0.001
		(0.008)	(0.008)		(0.005)	(0.005)		(0.007)	(0.006)
Change in BMI (age 5-15)		−0.000	−0.004		−0.003	−0.002		−0.000	0.003
		(0.008)	(0.007)		(0.006)	(0.005)		(0.007)	(0.006)
**Early adulthood experiences (Age 22, Round 7)**									
High interpersonal trust			−0.072∗∗			−0.017			0.019
			(0.030)			(0.016)			(0.042)
Big 5 Neuroticism (z-score)			0.160∗∗∗			0.077∗∗∗			0.269∗∗∗
			(0.022)			(0.015)			(0.020)
Grit Index (z-score)			0.033			−0.038∗∗			−0.015
			(0.027)			(0.015)			(0.027)
Food insecure			0.063∗∗∗			−0.001			0.066∗∗∗
			(0.024)			(0.017)			(0.024)
Increase in the price of food I buy			0.024			−0.003			0.044
			(0.024)			(0.015)			(0.035)
Job loss/source of income/family enterprise			0.066∗∗			0.051			0.019
			(0.030)			(0.032)			(0.023)
Theft or destruction of property			0.065∗∗			0.107∗∗			0.035
			(0.033)			(0.042)			(0.029)
Crops failed			0.144∗∗∗			0.006			0.096∗∗
			(0.047)			(0.016)			(0.042)
Illness of a family member			0.071∗∗∗			0.041∗∗∗			0.059∗∗∗
			(0.024)			(0.015)			(0.023)
Hours spent on caring/domestic chores past week			0.008∗			0.001			−0.001
			(0.005)			(0.003)			(0.005)
Spent 8-9 h sleeping past week			−0.011			−0.008			−0.029
			(0.023)			(0.015)			(0.021)
Disability affecting work/capacity take care of yourself			0.108			0.184			0.127
			(0.077)			(0.217)			(0.086)
Ever experienced IPV			0.065∗			0.055∗			0.181∗∗∗
			(0.035)			(0.031)			(0.027)
Ever been bullied over the internet			0.056			0.061			0.001
			(0.043)			(0.041)			(0.043)
Physical attacked due to conflict (includes attempt)			0.044∗						
			(0.026)						
Ever married or cohabitating			−0.014			−0.052∗∗			−0.014
			(0.040)			(0.026)			(0.030)
Underage marriage/cohabitation or teen parenthood			−0.065			0.089∗∗∗			0.068∗
			(0.054)			(0.030)			(0.038)
Ever drunk alcohol			−0.010			0.041∗			0.013
			(0.025)			(0.024)			(0.031)
Studying only			−0.017			0.004			0.083∗∗
			(0.035)			(0.023)			(0.036)
Working only			−0.036			0.023			0.028
			(0.031)			(0.023)			(0.027)
Neither working nor studying			−0.003			0.004			0.026
			(0.045)			(0.029)			(0.061)
Highest education level: Primary			−0.044			−0.050			0.138
			(0.032)			(0.043)			(0.125)
Highest education level: Secondary			−0.038			−0.045			0.227∗
			(0.033)			(0.039)			(0.128)
Highest education level: Higher			0.030			−0.052			0.195
			(0.053)			(0.040)			(0.130)
Change Wealth Index (age 1-22)			0.126			0.010			0.037
			(0.094)			(0.074)			(0.093)
Migrated (age 1-22)						−0.020			0.097∗∗∗
						(0.024)			(0.034)

Constant	0.086∗∗∗	0.150	−0.052	0.049∗	−0.047	−0.069	0.062	0.165	−0.239
	(0.025)	(0.128)	(0.148)	(0.028)	(0.080)	(0.105)	(0.039)	(0.139)	(0.173)

Observations	1231	1231	1231	1716	1716	1716	1558	1558	1558
R-squared	0.108	0.122	0.261	0.016	0.027	0.130	0.062	0.073	0.287
Round 1 Ethnicity FE	Yes	Yes	Yes	Yes	Yes	Yes	Yes	Yes	Yes
Round 7 Region FE	No	No	Yes	No	No	Yes	No	No	Yes

*Notes*: Results of columns (2) and (3) control for missing values for the mathematics score in Round 5. Results in columns (3) control for missing responses on reporting IPV, bullying online, physical attacks due to conflict and drinking alcohol. Robust standard errors are reported in parentheses. ∗∗∗p < 0.01, ∗∗p < 0.05, ∗p < 0.1.

#### 6.1.1 Sex

Our results for Peru and India are consistent with the literature, showing that females are more likely to exhibit symptoms of at least mild anxiety. This sex gap is substantially greater in Peru than in India, as indicated by the descriptive findings presented earlier, and the cross-country differences are statistically significant. While the estimated coefficient for sex in Peru remains relatively stable after accounting for adolescent experiences (column 2), it decreases considerably when controlling for young adulthood variables (column 3). This suggests that the anxiety gap by sex in Peru is mediated by other adult factors and experiences highlighted in the literature, which are included as covariates in the third specification—such as domestic violence and early motherhood.

In contrast, the estimated sex gap in India for anxiety remains largely unchanged when adding adolescent and early adulthood factors. In Ethiopia, there is no statistically significant sex gap for anxiety. Interestingly, when controlling for adulthood outcomes, the point estimate suggests males report higher rates of anxiety. It is plausible that this reflects the disproportional impact of the armed conflict on males relative to females, leading to a deterioration of males’ mental health.

#### 6.1.2 Early life conditions

We find that poorer caregivers' mental health is consistently associated with a higher probability of experiencing at least mild anxiety during early adulthood in Peru, and these cross-country differences are statistically significant throughout all specifications. The coefficient roughly halves when controlling for young adulthood covariates, suggesting the existence of mediating mechanisms in adulthood (column 3). In Ethiopia, caregivers’ mental health during early childhood is statistically insignificant. In India, we find no significant relationship when controlling for early life and adolescent experiences, and actually observe a negative association when controlling for young adulthood factors.

We find mixed results regarding the role of early-life socio-economic status, proxied by the household wealth index, caregiver literacy and, to some extent, area of residence. In Ethiopia, caregiver literacy is associated with a lower level of anxiety in columns (1) and (2), but the association loses statistical significance when young adulthood variables are included. Furthermore, the appendix test suggests coefficients are not different across countries. Regarding the household wealth index, in Peru, we observe that higher household wealth is associated with an increased likelihood of at least mild anxiety, contrasting with the existing literature, whereas the correlations are not significant for Ethiopia and India. The cross-country differences are statistically significant in columns (1) and (2), but not in column (3).

Finally, we observe that the prevalence of anxiety is higher among participants from urban areas in Ethiopia, while no such differences emerge in the other two countries, except in Peru when controlling for all factors (column 3). These cross-country differences are statistically significant throughout all specifications, suggesting that the urban gradient in anxiety is strongest in Ethiopia.

#### 6.1.3 Adolescent experiences

Our findings indicate that participants from India and Peru who reported stronger relationships with their parents during adolescence are less likely to experience anxiety in adulthood. In Peru, this result remains statistically significant even after accounting for young-adult regressors, whereas in India the relationship disappears after including these controls. In Ethiopia, the association is not statistically significant in any model. Despite these patterns, these coefficients do not differ significantly across countries.

Using socioemotional skills measured during adolescence, in column (2) we find that a higher sense of pride is associated with lower anxiety in Ethiopia, but not in India and Peru, and these cross-country differences are statistically significant. The relationship between pride and anxiety in Ethiopia reduces in magnitude but remains significant even after controlling for factors related to young adulthood (column 3), suggesting that pride developed during adolescence continues to predict mental health outcomes in young adulthood. In contrast, a higher sense of agency is associated with increased anxiety in Ethiopia—however, this result becomes not statistically significant when controlling for young adulthood factors. For agency, the differences in the coefficients across countries are not statistically significant in any specification.

Our findings also reveal a weak association between adolescent schooling and learning outcomes and adult anxiety. While higher mathematics test scores at age 15 are associated with lower anxiety in India, the magnitudes of the coefficients are close to zero, and the results lose statistical significance when accounting for adult covariates. We also find that spending more hours studying in adolescence is associated with a lower level of anxiety in Ethiopia, and only in this case are the cross-country differences statistically significant. All the other correlations for these two variables are not statistically significant. It is important to note that neither the correlation with school enrolment nor that with body mass index, or changes in body mass index, is significant in any country.

Overall, our findings show that positive social relationships and a sense of pride developed during adolescence are indicators of lower anxiety later in life, while the impact of learning and education seems limited.

#### 6.1.4 Early adulthood factors and experiences

Early adulthood predictors were measured concurrently with anxiety. In Ethiopia, our findings align with the literature, indicating that higher interpersonal trust in adulthood is associated with a lower likelihood of exhibiting symptoms of anxiety. This association does not differ significantly across countries.

The correlation between socio-emotional skills at age 22 and anxiety is stronger than that observed at age 15. Participants with higher neuroticism (emotional instability) scores at age 22 are significantly more likely to exhibit symptoms compatible with anxiety in all three countries. The coefficient is substantially higher for Peru, and the cross-country differences are statistically significant. Additionally, higher levels of grit are associated with a lower prevalence of anxiety in India, but not in the other countries. The cross-country differences are statistically significant, suggesting that the protective association with grit is more salient in India.

Food insecurity and exposure to shocks affecting household wealth are strongly correlated with mental health difficulties. First, participants living in food-insecure households in Ethiopia and Peru are more likely to exhibit symptoms compatible with at least mild anxiety, whereas the coefficient for India is virtually zero. Relatedly, exposure to crop failure is associated with increased anxiety in Ethiopia and Peru. In both cases, the cross-country differences are statistically significant, with the strongest associations concentrated in Ethiopia and Peru. Second, participants who reported the illness of a family member are more likely to experience anxiety across the three study countries—in this case, coefficients do not appear to differ across countries. Third, exposure to any of the other shocks observed (increases in food prices, job loss, theft or destruction of poverty) is significant for at least one country sample in at least one outcome.^15^ In these cases, the coefficients do not differ significantly across countries, and most of the point estimates are positive.

In relation to time use, in Ethiopia, more hours spent on domestic chores are correlated with higher anxiety, although the point estimate is small compared to other predictors The corresponding coefficients for India and Peru are also close to zero, and the differences across countries are not statistically significant. When considering the choice of working, studying, doing both or neither, in Peru, ‘studying only’ increases the prevalence of anxiety—compared to the alternative of both studying and working. Disability status does not predict anxiety, although the point estimates are relatively large across the three countries—the differences across countries are not statistically significant. The lack of statistical significance in this case might be linked to the very small proportions of participants for whom a disability is reported.

We observe that experiencing intimate partner violence (IPV) is consistently associated with higher levels of anxiety across the three study countries. The point estimate for Peru triples that observed for Ethiopia and India, and the cross-country differences are statistically significant. In Ethiopia, individuals reporting physical attacks are significantly more likely to experience mild anxiety. These results align with prior research using Young Lives data, which documented the detrimental effects of armed conflict on the mental health of young people in Ethiopia,^16^^,^ 17 (Favara et al., 2022). These results underscore the negative impact of violence on mental health outcomes.^18^

Analysing marriage and parenthood, we find that being married or cohabiting is associated with a lower level of anxiety in India, though these coefficients do not differ systematically across the three countries. However, participants who marry, cohabit, or become parents at a young age face a higher risk of experiencing anxiety in India and Peru, and these cross-country differences are statistically significant. Previous research using Young Lives data has documented that teen marriage, cohabitation, and parenthood are correlated with a lower likelihood of completing high school, reduced agency, and lower life satisfaction and self-esteem (Briones & Porter, 2019). Importantly, our results remain robust when controlling for educational attainment and adolescent agency and pride. Finally, our results on alcohol align with the literature, showing that alcohol consumption is associated with higher anxiety in India. Although the associations for India are particularly pronounced, the differences across countries are not statistically significant.

We do not observe strong evidence of an association between educational attainment in young adulthood and anxiety across countries; however, in Peru, having completed secondary school is associated with higher anxiety. Finally, considering the role of changing circumstances over the life-course, we document that migration between ages one and 22 is associated with higher levels of anxiety in Peru. Although migration does not significantly predict mental health outcomes in India, it may be correlated with other variables already included in the model, such as exposure to shocks, time use, and family formation. In fact, the coefficient for Ethiopia is not defined due to collinearity with other factors already included in the model.

In general, results for depression are like those observed for anxiety; the key difference is that only a subset of the factors that predict anxiety also predict depression. Considering the full specification (column 3), we highlight the following differences (Table A.6 in the Online Appendix). First, there is no sex gap for depression in India—as there was for anxiety. Second, caregiver's mental health does not play a role for adult depression in Peru. Third, there is no evidence of higher levels of depression for those born in urban areas in Ethiopia or Peru, nor a relationship between higher pride or higher interpersonal trust and lower depression in Ethiopia. Fourth, migration, which matters to predict anxiety in Peru, is not relevant for depression in the same country. Despite these differences, it is important to highlight that exposure to recent shocks and exposure to violence—including conflict—seem to be similarly important to predict both anxiety and depression.

In one notable discrepancy, underage marriage and parenthood in Ethiopia are associated with lower levels of depression—but to higher levels of anxiety in India and Peru. Previous literature suggests that economic hardship is a key driver of early marriage in Ethiopia, the poorest country in our sample, and is often used as a strategy to alleviate financial strain and improve economic opportunities, particularly for girls (Gavrilovic et al., n.d.). Consequently, this finding may partly reflect an improvement in economic circumstances compared to prior conditions. However, alternative explanations may exist for this association.

### 6.2 Changes in mental health between 2020 and 2024: value-added specifications

To reduce bias from unobserved individual heterogeneity, we next estimate value-added specifications, i.e., we incorporate anxiety symptoms measured in August-October 2020 into the specification for anxiety, and restrict covariates to predictors measured in 2023. Results are reported in Table 2, and results for depression are reported in Table A.7 in the Online Appendix.

**Table 2:** Valued-added regressions on anxiety prevalence, (at least mild, %).

	Ethiopia	India	Peru
	(1)	(2)	(3)
At least mild depression (2020)	0.005	0.048	0.151∗∗∗
	(0.033)	(0.030)	(0.026)
Female	−0.033	0.041∗∗	0.064∗∗
	(0.025)	(0.019)	(0.026)
**Early adulthood experiences (Age 22, Round 7)**			
High interpersonal trust	−0.070∗∗	−0.019	0.040
	(0.030)	(0.016)	(0.044)
Big 5 Neuroticism (z-score)	0.150∗∗∗	0.076∗∗∗	0.241∗∗∗
	(0.022)	(0.014)	(0.022)
Grit Index (z-score)	0.037	−0.040∗∗∗	−0.029
	(0.027)	(0.015)	(0.028)
Food insecure	0.054∗∗	0.004	0.056∗∗
	(0.023)	(0.017)	(0.024)
Increase in the price of food I buy	0.027	−0.009	0.009
	(0.023)	(0.015)	(0.038)
Job loss/source of income/family enterprise	0.083∗∗∗	0.045	0.023
	(0.030)	(0.032)	(0.024)
Theft or destruction of property	0.072∗∗	0.097∗∗	0.044
	(0.034)	(0.042)	(0.031)
Crops failed	0.134∗∗∗	0.013	0.066
	(0.048)	(0.015)	(0.044)
Illness of a family member	0.069∗∗∗	0.037∗∗∗	0.041∗
	(0.025)	(0.014)	(0.024)
Hours spent on caring/domestic chores past week	0.006	0.001	0.001
	(0.005)	(0.003)	(0.005)
Spent 8-9 h sleeping past week	−0.026	−0.010	−0.038∗
	(0.023)	(0.015)	(0.022)
Disability affecting work/capacity take care of yourself	0.121	0.222	0.122
	(0.080)	(0.221)	(0.090)
Ever experienced IPV	0.042	0.055∗	0.155∗∗∗
	(0.035)	(0.031)	(0.029)
Ever been bullied over the internet	0.053	0.060	−0.005
	(0.042)	(0.041)	(0.047)
Physical attacked due to conflict (includes attempt)	0.038		
	(0.026)		
Ever married or cohabitating	−0.005	−0.054∗∗	−0.027
	(0.041)	(0.026)	(0.032)
Underage marriage/cohabitation or teen parenthood	−0.076	0.088∗∗∗	0.055
	(0.052)	(0.030)	(0.041)
Ever drunk alcohol	0.007	0.043∗	0.044
	(0.025)	(0.024)	(0.031)
Studying only	−0.002	0.003	0.081∗∗
	(0.035)	(0.024)	(0.037)
Working only	−0.027	0.021	0.003
	(0.031)	(0.022)	(0.027)
Neither working nor studying	0.011	0.000	−0.023
	(0.045)	(0.029)	(0.066)
Highest education level: Primary education	−0.005	−0.059	0.146
	(0.031)	(0.042)	(0.162)
Highest education level: Secondary education	0.014	−0.028	0.207
	(0.028)	(0.038)	(0.159)
Highest education level: Higher education	0.104∗∗	−0.043	0.162
	(0.049)	(0.039)	(0.160)

Constant	0.035	0.064	−0.093
	(0.056)	(0.047)	(0.165)

Observations	1183	1711	1376
R-squared	0.237	0.120	0.289
Round 1 Ethnicity FE	No	No	No
Round 7 Region FE	Yes	Yes	Yes

*Notes:* Robust standard errors are reported in parentheses. Results control for missing responses on reporting IPV, bullying online, physical attacks due to conflict and drinking alcohol. ∗∗∗p < 0.01, ∗∗p < 0.05, ∗p < 0.1.

Our findings reveal that mental health symptoms during the pandemic are significant predictors of both anxiety and depression in 2023/24 in Peru and depression only in India. In Ethiopia, there is no statistically significant association, and the point estimates are also close to zero for both anxiety and depression, which is likely due to the devastating effects of the civil war, which may have disrupted prior mental health trajectories and rendered mental health before the conflict less predictive of mental health after the war. Sex differences are consistent with Section 7.1, with females reporting significantly greater increases in anxiety in India and Peru, and depression in Peru.

Even after controlling for lagged mental health, our results largely confirm prior findings for young adulthood variables. Higher neuroticism is consistently associated with increases in anxiety (and depression) in all three countries, while interpersonal trust is associated with decreased anxiety (and depression) in Ethiopia. In India, higher levels of grit remain associated with decreased anxiety (and depression in both India and Peru). Food insecurity continues to be correlated with increases in anxiety (and depression) in Ethiopia and Peru. Job or income loss is similarly correlated with increased anxiety in Ethiopia (and depression in both Ethiopia and Peru). Theft or destruction of property is also associated with higher anxiety in Ethiopia as well as India (and depression in Ethiopia only). Crop failure continues to predict increased anxiety in Ethiopia (and depression in both Ethiopia and Peru), and illness of a family member is associated with higher anxiety across the three countries (and depression in Ethiopia and India only).

Violence also remains an important determinant of mental health. Participants who report experiencing IPV are significantly more likely to experience increases in anxiety in India and Peru. However, no significant association is observed in Ethiopia (where, instead, there is an association with depression as in Peru). Exposure to physical attacks during the Ethiopian civil conflict is no longer associated with increased anxiety. Patterns for marriage and cohabitation, as well as alcohol consumption, are largely consistent with earlier findings.

Taken together, the results presented in Table 2 are consistent with those in Table 1. While the analysis remains observational, this alignment is encouraging and suggests underlying causal mechanisms, and the possibility that the significant predictors identified in our value-added regressions may reflect causal relationships rather than mere associations.

Analysing predictors separately by sex (Table A.8 and Table A.9), we find notable differences in how various factors influence mental health in young adulthood. For example, social factors, such as interpersonal trust and marital status, appear to play a greater role in predicting female anxiety prevalence. In contrast, economic stressors, such as theft or destruction of property, seem more relevant in predicting male anxiety. In contrast, predictors such as neuroticism do not seem to have a differential impact on both anxiety and depression by sex. Further research, including mixed-methods approaches, is needed to understand these differences.

## 7 Discussion

The key contribution of our paper is to present, to our knowledge, the first multi-country investigation of early-life, adolescent, and young-adult factors associated with adulthood mental health in the developing world, using detailed, comparable data spanning over two decades. Drawing on rich, individual-level panel data from Ethiopia, India, and Peru, we examine how sex, early childhood conditions, and experiences during adolescence and young adulthood are associated with symptoms of anxiety and depression at age 22 — also referred to as the “impressionable years.” — a critical time for the onset of mental health issues (Patel et al., 2018), with long-term and even intergenerational consequences (Burcusa & Iacono, 2007).

Our evidence suggests that reported symptoms of anxiety and depression vary significantly across countries and over time, with Peru exhibiting the highest prevalence, Ethiopia showing post-conflict deterioration, and India remaining relatively stable. Early-life conditions such as caregiver mental health and household wealth are linked to later mental health, particularly in Peru. During adolescence, strong parent-child relationships and a sense of pride are associated with improved mental health, while academic performance shows weaker associations. In young adulthood, emotional instability—neuroticism—is consistently associated with worse mental health, likely reflecting its association with poor stress regulation. In comparison, grit is associated with better mental health in India. Sex disparities are substantial, with females reporting significantly worse mental health in Peru and India.

Economic shocks (e.g., job loss, food insecurity, crop failure) and experiences of violence, including intimate partner violence and conflict-related violence in Ethiopia, emerge as strongly associated with poor mental health. However, these patterns should be interpreted in light of the Young Lives study's pro-poor sampling design, which may shape the observed associations. Participants' limited ability to buffer shocks likely amplifies their vulnerability and the mental health effects of adverse events. As such, while the findings provide valuable insights into vulnerable populations, further work is needed to assess their generalisability to wealthier groups.

While in some cases the magnitude of the associations is similar across countries, in other cases they differ substantially. Focusing on adult anxiety for simplicity, we document that the associations with sex, caregiver's mental health, area of residence and, to a lesser extent, household's wealth index vary across countries. For example, we find that higher household wealth is associated with poorer mental health in Peru, whereas the correlations are not significant for Ethiopia and India. One possible explanation is that individuals from wealthier backgrounds may face heightened pressure to maintain their socioeconomic status and achieve high expectations, which can contribute to increased anxiety.^19^ This effect may be particularly pronounced after the COVID-19 pandemic, which disproportionately impacted Peru and may have disrupted the trajectories of wealthier young adults in particular, creating a mismatch between their aspirations and reality. Considering the role of adolescence and early adulthood factors and experiences, we further document that the associations of adult anxiety with sense of pride, study hours, neuroticism, grit, food insecurity, crop failure, IPV, underage marriage and parenthood and studying also vary across countries. These differences in magnitude likely reflect the distinct recent contexts of the three countries, including Peru's persistently higher anxiety levels, Ethiopia's conflict-related and agricultural shocks, and the relatively more stable mental health profile observed in India.

While the use of standardized survey instruments and a comparable sampling design across the three countries mitigates concerns that observed differences arise from poor harmonisations or sampling inconsistencies, the contextual interpretations we offer are best viewed as plausible hypotheses grounded in contextual knowledge, rather than definitive causal mechanisms. Moreover, although the analysis draws on rich longitudinal data, the patterns over time in anxiety and depression are primarily descriptive. Because we follow a single birth cohort observed between ages 19 and 22, changes in mental health over this period may partly reflect a combination of developmental processes during young adulthood and exposure to contemporaneous shocks. Our value-added specifications, which condition on prior mental health, help account for baseline vulnerability and symptom persistence, thereby strengthening the interpretation of associations observed in young adulthood. However, they do not fully eliminate the potential influence of unobserved, time-varying factors, and therefore, the findings should be interpreted as associations rather than causal effects.

A comparison between our findings and those from high-income countries (HICs) (Cadman et al., 2024; Currie & Morgan, 2020) shows both consistent patterns in the predictors of mental health and unique, context-specific vulnerabilities. A clear finding is the significant gender disparity in mental health outcomes: we observe that females report notably worse mental health in Peru and India, consistent with HIC evidence that girls and women generally exhibit a higher prevalence of internalizing symptoms, psychosomatic complaints, and generally score less favourably on measures of emotional well-being. Likewise, the importance of supportive relationships holds in both contexts — our results linking positive parent–child relationships to better adult mental health align with HIC evidence that emphasizes parental communication and emotional support as key protective factors.

However, several differences also emerge. Whereas HIC studies (Cadman et al., 2024) consistently show that children from more deprived households experience worse mental health from early childhood onward, our results indicate mixed links for early-life wealth — including an association between higher childhood wealth and poorer mental health in Peru. Additionally, our findings emphasize the importance of acute stressors, such as economic shocks and exposure to violence, as key factors of poorer mental health, highlighting vulnerabilities caused by economic and political instability that are less evident in the general HIC cohorts examined in comparable studies.

## 8 Conclusions

By leveraging the extensive information collected during the Young Lives study, we provide new insights into how early-life and adolescent experiences, as well as more recent factors, correlate with mental health during early adulthood.

Our findings underscore the potential critical role of human and social capital, as well as healthy parental relationships in early life, in shaping mental health in young adulthood. They emphasize the importance of early interventions that support both parents and children, laying a foundation for promoting mental well-being throughout the life course.

Non-cognitive skills and personality traits during adolescence and adulthood also emerge as important predictors of mental health. However, their relationships vary by context, suggesting that cultural norms and socio-economic conditions shape the impact of psychological resilience traits.

Consistent with a growing literature linking economic shocks to mental health, our findings show that exposure to adverse events is strongly correlated with poorer mental health. The association between mental health and violence is similarly profound. IPV consistently predicts worse mental health outcomes across all study settings. In Ethiopia, exposure to physical violence during the recent civil conflict is also linked to elevated symptoms of anxiety and depression, underscoring the deep and lasting psychological impact of armed conflict.

While we do not establish causal relationships, our findings identify key factors associated with mental health across the life course. These results highlight the need to identify and support at-risk individuals through targeted interventions at different developmental stages. Although some risk factors are consistent across settings, many are context-specific, underscoring the importance of tailoring mental health policies to local economic, social and cultural conditions. In addition, the persistent sex gap reinforces the need to integrate a sex-sensitive lens into program and policy design. Ultimately, improving mental health and resilience will require holistic, life-course approaches that are both context-aware and equity-focused.

## CRediT authorship contribution statement

**Marta Favara:** Writing – review & editing, Writing – original draft, Supervision, Project administration, Methodology, Investigation, Funding acquisition, Conceptualization. **Richard Freund:** Writing – review & editing, Writing – original draft, Methodology, Conceptualization. **Juliana Quigua:** Writing – review & editing, Writing – original draft, Software, Methodology, Formal analysis, Data curation, Conceptualization. **Alan Sánchez:** Writing – review & editing, Writing – original draft, Supervision, Project administration, Methodology, Investigation, Conceptualization.

## Data statement

Survey data from the Young Lives study previous to Round 7 is available via the UK Data Service (https://beta.ukdataservice.ac.uk/datacatalogue/series/series?id=2000060). The data for Round 7, which is main source of data of this paper, will be publicly available in the same portal by 2026.

## Ethical statement

Ethics approvals for the Young Lives Study were obtained by the Social Sciences and Humanities Inter-Divisional Research Ethics Committee (SSH IDREC) at the University of Oxford: in October 2022 (Ref.: SSH/ODID_C1A_22_088); in April 2023 (Ref.: R85604/RE001); in February 2024 (Ref.: C1A_23_002); and in March 2024 (Ref.: R85604/RE001). The study was also approved in each study country: in Ethiopia, approval as granted by the National Research Ethics Review Board of the Ethiopian Ministry of Education (Ref.:17/152/702/23) and the College of Education and Behavioural Studies Institutional Review Committee (Ref.: CEBS_IRC/009/2023); in India by the Centre for Economic and Social Studies in Hyderabad and the Institutional Ethics Committee for Biomedical Research at the Institute of Genetics & Hospital for Genetics Diseases (Ref.:766/1/IG/IECBR/2023); and IN Peru by the Instituto de Investigacion Nutricional (Ref.:N°180-2002/CIEI-IIN)

## Declaration of generative AI and AI-assisted technologies in the writing process

During the preparation of this work, the author(s) used Gemini and Grammarly in order to improve language and readability. After using this tool/service, the author(s) reviewed and edited the content as needed and take(s) full responsibility for the content of the publication.

## Funding

Thanks to the UK's 10.13039/501100020171Foreign, Commonwealth and Development Office (10.13039/501100020171FCDO) for funding Young Lives at Work, enabling this research, and to the 10.13039/100010269Wellcome Trust for supporting Young Lives research on mental health. The funders had no role in the design, interpretation, or writing-up of the study or in the decision to submit the study for consideration for publication.

## Declaration of competing interests

None of the authors has a competing interest to declare.

## Data Availability

The Young Lives data are publicly available through the UK Data Service. The Round 7 data are not in the public domain yet. They will release early next year

## Footnotes

1 https://vizhub.healthdata.org/.

2 In this study, we use sex instead of gender since we rely on a binary categorization (male/female) that is designated at birth. However, we expect the differences to reflect socially constructed roles and different life experiences related to the gender associated with the sex assigned at birth.

3 In 2014, Andhra Pradesh, which had a population of 84 million in 2011, was divided into two separate states—Telangana and Andhra Pradesh. In the paper we will often refer to results for ‘India’ or ‘the Indian sample’; readers are requested to keep in mind that the sample is exclusively based in this one state.

4 The ‘Older Cohort’, born in 1994/95, were already aged eight in the first survey round, which limits the availability of information on their early childhood conditions. For this reason, we have excluded them from the analysis. Additionally, Vietnam has been excluded from this paper because the seventh survey round—our primary source of outcome data—was not conducted in the country due to a change in governmental procedures around the international transfer of personal data. For more information on Young Lives, visit:https://www.younglives.org.uk/.

5 The ninth item of the PHQ, which addresses suicidal ideation, was excluded because of ethical concerns regarding the ability to provide adequate support. The instruments were also modified slightly for use in a phone-based survey. Respondents were first asked if they were alone during the call; if not, they were encouraged to move to a quiet location and/or switch off the phone speaker. For each symptom, individuals were initially asked whether it had occurred at all in the preceding 14 days (Yes/No), and only if they responded ‘Yes’ were follow-up questions posed about frequency. These adaptations were tested in a pilot before data collection, and the modules were placed at the end of the survey.

6 The PHQ-8 scale has been validated in several countries, including Peru. In India and Ethiopia, the PHQ-9 scale has been validated.

7 The cutoffs for anxiety are as follow: “minimal” (score below 4), “mild” (score between 5 and 9), “moderate” (score between 10 and 14), and “severe” (score above 15). For depression, the cutoffs are: “minimal” (score below 4), “mild” (score between 5 and 9), “moderate” (score between 10 and 14), “moderately severe” (score between 15 and 19) and “severe” (score above 20).

8 In Figure A.1 in the Appendix, we report the anxiety trend of the Ethiopian sample when including participants in Amhara who were interviewed via phone survey. We find that the trends are very similar with or without the phone survey sample.

9 However, at least part of this decline might be due to the fact that Young Lives was not able to interview participants in Tigray in 2021.

10 Comparisons with OECD countries further contextualize these findings. Using similar screening tools (PHQ-4, a combination of PHQ-2 and GAD-2), research conducted in OECD countries indicates that over 25% of individuals in 15 OECD countries exhibited symptoms compatible with anxiety and depression during 2020-2021 (OECD, 2021).

11 Additional information about the measurement of the explanatory variables is reported in Appendix A.1.

12 In Ethiopia, we classify participants in ethnic groups Amhara, Oromo, Tigrigna, 'Others'; in India, we consider the caste categories Scheduled Castes, Scheduled Tribes, Backward Classes, and other groups with or without caste affiliation as 'Others'; in Peru, we classify participants based on whether their mother spoke Spanish or another language.

13 Previous literature highlights that anxiety is more stable and trait-like compared to depression, which tends to be more episodic (Prenoveau et al., 2011).

14 To assess the robustness of our results in Ethiopia to conflict-related missing data, in Table A.4 we re-estimate the specifications incorporating covariates from early childhood and adolescence, for the full Ethiopia sample, i.e. including the participants from the two sites of Amhara who were interviewed via phone. We anticipate results for this extended sample are largely unchanged. However, we are unable to test the robustness of results using young adulthood regressors, as the phone survey did not include questions on personality traits, exposure to violence, or risky behaviors due to its shorter format.

15 In Ethiopia, participants who reported losing their jobs or their primary source of income exhibit increased anxiety. Similarly, exposure to theft or destruction of property is associated with higher anxiety in Ethiopia and India.

16 Due to the recent armed conflict, this data was collected exclusively in Ethiopia using audio computer-assisted self-interviewing (ACASI) technology. For further details, see van Russdorf et al. (2024).

17 Our results might represent a lower bound of the association between exposure to physical violence due to the conflict and mental health outcomes, as some of the attrition observed in the last visit to the Ethiopian sample might be due to the conflict.

18 However, it is important to acknowledge that our data capture exposure to armed conflict and other forms of violence only at a single point in young adulthood, and only in Ethiopia for conflict-related violence. We are therefore unable to account for the timing, severity, or duration of exposure across earlier developmental stages, or to assess whether the magnitude of these associations differs when violence occurs in childhood or adolescence. As such, the observed associations should be interpreted as reflecting contemporaneous exposure at this specific age, recognizing that developmental timing and chronicity may shape vulnerability to violence differently across the life course. Future work with repeated measures of violence exposure across stages would be better equipped to unpack these dynamics.

19 For instance, research has evidenced that individuals with higher socioeconomic status exhibit higher association between job pressure and anxiety (Koltai & Schieman, 2015).
