# Supplementary material for: Factors associated with mental health among young adults: Cross-country longitudinal evidence from Ethiopia, India, and Peru
Source: SSM Popul Health. 2026 Mar 24;34:101911. doi: 10.1016/j.ssmph.2026.101911 (PMC13087745; doi:10.1016/j.ssmph.2026.101911)
Supplement: Multimedia component 1 [file mmc1.docx]

**Online Appendix**

**Appendix A.1 Variables included in the analysis**

This appendix details the construction of the covariates used in the analysis. Table A.1. provides descriptive statistics for the variables used in the analysis.

**Table A.1.** Descriptive statistics

|  | Ethiopia | India | Peru |
| --- | --- | --- | --- |
| **Round 1: 2002 (age 1)** |  |  |  |
| Index participant is a woman (%) | 49.7 | 45.5 | 51.0 |
| Caregiver is literate (%) | 28.4 | 34.6 | 73.0 |
| Area of residence is urban (%) | 36.2 | 22.4 | 69.3 |
| Caregiver’s Mental Health (0-20 score, higher score = worse mental health) | 5.8 | 5.5 | 5.7 |
| Wealth index - Bottom tercile (%) | 31.5 | 34.0 | 32.7 |
| Wealth index - Middle tercile (%) | 33.6 | 35.3 | 33.1 |
| Wealth index -Top tercile (%) | 34.8 | 30.7 | 34.2 |
| **Round 5: 2006 (age 15)** |  |  |  |
| Parent-child relationship Index (1-32 score, higher score = better relationship) | 26.3 | 27.8 | 25.0 |
| Pride Index | 21.0 | 17.4 | 23.4 |
| Agency Index | 18.9 | 17.3 | 17.2 |
| Math Test (% Correct) * | 30.0 | 33.2 | 37.1 |
| Missing (%) | 4.7 | 2.9 | 0.0 |
| Study Hours (Last Week) | 7.3 | 9.9 | 9.0 |
| Enrolled in School | 0.9 | 0.9 | 1.0 |
| BMI | 17.0 | 17.9 | 21.7 |
| **Round 7: 2023/24 (age 22)** |  |  |  |
| Reports high interpersonal trust (%) | 16.7 | 61.1 | 5.4 |
| Neuroticism score (0-40 score) | 19.3 | 18.0 | 22.7 |
| Grit Index (1-60 score) | 40.9 | 43.0 | 40.5 |
| Food insecure (%) | 73.6 | 79.8 | 58.9 |
| Self-reported shock: Job loss/source of income/family enterprise (%) | 29.1 | 7.5 | 47.9 |
| Self-reported shock: Theft or destruction of property (%) | 17.5 | 4.5 | 17.7 |
| Self-reported shock: Crops failed (%) | 8.4 | 31.4 | 7.8 |
| Self-reported shock: Illness of a family member (%) | 30.3 | 34.9 | 43.2 |
| Self-reported shock: Increase in the price of food that I buy (%) | 82.7 | 67.0 | 90.4 |
| Hours spent on caring/domestic chores past week | 3.6 | 3.7 | 3.2 |
| Disability affecting work/capacity take care of yourself (%) | 2.9 | 0.2 | 2.0 |
| Ever experienced IPV * (%) | 11.7 | 10.2 | 26.3 |
| Missing (%) | 0.7 | 1.5 | 0.1 |
| Ever bullied over the internet * (%) | 8.2 | 5.2 | 6.9 |
| Missing (%) | 9.8 | 12.1 | 1.1 |
| Physical attacked due to conflict (includes attempt) (%) | 32.0 | . | . |
| Missing (%) | 7.7 | 0.0 | 0.0 |
| Ever married or cohabitating (%) | 12.2 | 24.6 | 27.7 |
| Underage marriage/cohabitation or teen parenthood (%) | 5.6 | 10.9 | 15.2 |
| Ever drunk alcohol * (%) | 32.1 | 14.2 | 80.1 |
| Missing (%) | 7.3 | 6.8 | 1.8 |
| Wealth index - Bottom tercile (%) | 34.2 | 33.6 | 32.8 |
| Wealth index - Middle tercile (%) | 31.8 | 34.7 | 33.8 |
| Wealth index-Top tercile (%) | 34.0 | 31.8 | 33.4 |
| Studying only (%) | 18.8 | 16.0 | 11.4 |
| Working only (%) | 50.7 | 50.3 | 49.5 |
| Neither working nor studying (%) | 16.2 | 20.6 | 5.1 |
| Highest educational degree - No certificate (%) | 21.5 | 5.1 | 0.4 |
| Highest educational degree - Primary education (%) | 17.2 | 10.8 | 7.4 |
| Highest educational degree - Secondary Education (%) | 53.9 | 50.6 | 78.1 |
| Highest educational degree - Higher education (%) | 7.4 | 33.5 | 14.1 |
| Spent 8-9 hours sleeping past week (%) | 64.0 | 71.2 | 49.0 |
| **Observations** | 1231 | 1716 | 1558 |
| *Notes*: In each country, descriptive statistics are presented for a balanced sample of individuals who were present in the phone surveys as well as the seventh round of data collection. The mathematics score in Round 5 and the variables indicating if participants ever drank alcohol, ever experienced IPV, or were ever bullied over the internet have fewer observations since some participants did not complete the test or refused to answer these questions, respectively. We present the scores (or prevalence) of these variables and the percentage of participants with missing information below each variable. | | | |

**Variable descriptions**

Sex (Age 1, Round 1)

Sex is a binary categorization (male/female) reported by a household member when the participant was one year old.

Early life conditions (Age 1, Round 1)

- Caregiver is literate: takes the value of 1 if the participant’s caregiver is literate in any language (in Ethiopia and India) or the mother is literate in Spanish (in Peru).
- Maternal mental health: A score ranging from 0 to 20 based on the Self-Report Questionnaire (SRQ-20) (Beusenberg et al., 1994), where higher scores indicate poorer mental health. It was asked to the participants’ mothers in Peru and the primary caregivers in Ethiopia and India. The scale includes the following items, each scored as one if the symptom is present:
  1. Did you often have headaches?
  2. Was your appetite poor?
  3. Did you sleep badly?
  4. Were you easily frightened?
  5. Did your hands shake?
  6. Did you feel nervous, tense or worried?
  7. Was your digestion poor?
  8. Did you have trouble thinking clearly?
  9. Did you feel unhappy?
  10. Did you cry more than usual?
  11. Did you find it difficult to enjoy your daily activities?
  12. Did you find it difficult to make decisions?
  13. Did your daily work suffer?
  14. Were you unable to play a useful part in life?
  15. Did you lose interest in things?
  16. Did you feel you were a worthless person?
  17. Were things so bad that you felt that you just couldn’t go on?
  18. Did you feel tired all of the time?
  19. Did you have uncomfortable feelings in your stomach
  20. Were you easily tired?
- Wealth index: A score between 0 and 1, with a higher value indicating a higher socio-economic status. It is constructed based on three dimensions: housing quality, access to services, and ownership of consumer durables (Briones, 2017). For descriptive purposes, we report the percentage of participants in each tercile, calculated using the complete Round 1 sample within each country.
- Urban: takes the value of 1 if the participant’s family lives in an urban area, and 0 otherwise. The definition of urban/rural areas is country-specific and is defined as a function of the population within a given geographical unit.

Adolescent experiences (Age 15, Round 5)

- Math Test: Percentage of correct answers based on the mathematics tests administered to participants. Although the tests differed across countries (31 items in India and Peru, and 30 in Ethiopia), a larger number of common items was included in all countries. For further details, please refer to Revollo and Scott (2022).
- Pride Index: A score between 1 and 30 to assess participants' self-perceived value for themselves. It is based on the scale developed by Rosenberg (1965) and used in Young Lives after several adjustments (Yorke and Ogando Portela, 2018). A higher value indicates higher pride. The scale consists of the following items, each rated on a 5-point scale based on the participant's level of agreement (Strongly Disagree, Disagree, More or Less, Agree, Strongly Agree) (negative items are recoded):

1. I am proud of my clothes.
2. I am often embarrassed because I do not have the right books, pencils, and other equipment for school.
3. I am proud of my shoes or of having shoes
4. I am proud that I have the correct uniform.
5. I am proud of the work I have to do.
6. I feel my clothing is proper for all occasions.

- Agency Index: A score between 1 and 25 to assess participants' own beliefs about whether outcomes are due to their own efforts or the result of luck, fate, or the intervention of others (Maddux, 1991), i.e., a high sense of agency. A higher value indicates higher agency. The scale consists of the following items, each rated on a 5-point scale based on the participant's level of agreement (Strongly Disagree, Disagree, More or Less, Agree, Strongly Agree) (negative items are recoded):

1. If I try hard, I can improve my situation in life
2. Other people in my family make all the decisions about how I spend my time [recoded to positive]
3. I like to make plans for my future studies and work
4. If I study hard at school, I will be rewarded by a better job in the future
5. I have a choice about the work I do—I must do this sort of work

We present the average score of Agency and Pride in Table A.1 for descriptive purposes. We included the standardized z-score of both variables in the analysis.

- Parent-child relationship Index: A score between 1 and 32, based on the Marsh Self-Description Questionnaire II (Marsh and O’Neill, 1984). A higher value indicates a better self-perceived relationship between the participants and their parent(s). The scale consists of the following items, each rated on a 4-point scale based on the participant's level of agreement (Strongly Disagree, Disagree, Agree, Strongly Agree):
  1. I like my parents
  2. My parents like me
  3. My parents and I spend a lot of time together
  4. I get along well with my parents
  5. My parents understand me
  6. If I have children of my own, I want to bring them up like my parents raised me
  7. My parents are easy to talk to
  8. My parents and I have a lot of fun together
- Schooling variables: ‘Enrolment status’ is a binary variable that takes the value of 1 if a participant is enrolled at school at the moment of the interview, and 0 otherwise. ‘Study hours’ measures the number of hours the participant dedicates to study during a typical day (from Monday to Friday). This variable comes from the self-reported Time Allocation module.
- Body mass index (BMI): Defined as weight in kilograms divided by height in meters squared. We use the BMI z-score, which indicates how an adolescent's BMI compares with the average for their age and sex, based on the World Health Organization reference curves. To compare the change in BMI, we calculate the difference between BMI z-score at age 15 and BMI z-score at age 1. A positive value means the participant’s BMI z-score improved over time.

Early adulthood experiences (age 22, Round 7)

- Neuroticism score: A score between 0 and 40, based on the items of the Big Five Inventory (John & Srivastava, 1999), to assess the level of proneness to emotional instability and distress of the participants. A higher value indicates higher self-esteem. The scale consists of the following items, each rated on a 5-point scale based on the participant's level of agreement (Strongly Disagree, Disagree, More or Less, Agree, Strongly Agree):

1. I am someone who is depressed, blue
2. I am someone who is relaxed, handles stress well
3. I am someone who can be tense
4. I am someone who worries a lot
5. I am someone who is emotionally stable, not easily upset
6. I am someone who can be moody
7. I am someone who remains calm in tense situations
8. I am someone who gets nervous easily

For descriptive purposes, we present the average score in Table A.1. In the analysis, we included the standardized z-score.

- Grit Index: A score between 1 and 60 to assess, developed by Duckworth et al. (2007) to assess the level of perseverance and commitment for long-term goals of the participants. A higher value indicates higher self-esteem. The scale consists of the following items, each rated on a 5-point scale based on the participant's level of agreement (Strongly Disagree, Disagree, More or Less, Agree, Strongly Agree):

1. New ideas and projects sometimes distract me from previous ones
2. I have been obsessed with a certain idea or project for a short time but later lost interest
3. I often set a goal but later choose to pursue a different one
4. I have difficulty maintaining my focus on projects that take more than a few months
5. My interests change from year to year
6. I become interested in new pursuits every few months
7. Setbacks don’t discourage me
8. I am a hard worker
9. I finish whatever I begin
10. I am diligent
11. I have achieved a goal that took years of work.
12. I have overcome setbacks to conquer an important challenge.

For descriptive purposes, we present the average score in Table A.1. In the analysis, we included the standardized z-score.

- High interpersonal trust: it takes the value of 1 if the participant answers “Most people in my neighborhood can be trusted” instead of “You can’t be too careful/You cannot trust people at all”.
- Food insecure: it takes the value of 1 if the participant reports at least mild food insecurity. This indicator comes from a modified version of the Household Food Insecurity Access Scale (HFIAS) (Coates et al., 2007). Key changes included using a 12-month recall period rather than the standard 1-month recall and selecting six items from the original nine-item scale, with corresponding methodological adjustments. Table A.2 presents the items included in the food insecurity measure, and Table A.3 presents the methodology for calculating it.

**Table A.2.** *Items includes to create the food insecurity measure*

| **Item (numbers are assigned following the HFIAS numbering)** | **Included in the calculation** |
| --- | --- |
| Which of the following statements best describes the food situation at your home in the last twelve months? | No |
| 1. In the past 12 months, did you ever worry that your household would run out of food before you get money to buy or could acquire more? | Yes |
| 1. Were you or any household member not able to eat the kinds of foods you want because of lack of money? (For example, no meat, no fish, no fruit, no sweet) | No |
| 1. Did you or any household member have to eat a limited variety of foods due to lack of money? (For example, only rice and one vegetable, no meat) | Yes |
| 1. Did you or any household member have to eat some foods that you did not want to eat because of a lack of money to obtain other types of food? (for example, wild foods, immature crops, broken rice, discarded food) | No |
| 1. Did you or any household member have to eat less (portion size) in a meal than you wanted because there was not enough food? | Yes |
| 1. Did you or any household member have reduced the number of meals eaten a day because there was not enough food? (for example skip breakfast or lunch) | Yes |
| 1. Was there ever no food to eat in your household because of lack of money to get food? | Yes |
| 1. Did you or any household member go to sleep at night hungry because there was not enough food? | Yes |
| 1. Did you or any household member go a whole day and night without eating anything because there was not enough food? | No |
| Were the children in the household also affected? | No |

**Table A.3***. Food insecurity definition: adjusted HFIAS scale*

| **Household Food Insecurity Access category** | **HFIAS** | **YL HFIAS (follows HFIAS numbering)** |
| --- | --- | --- |
| Food secure | [(Q1=0 or Q1=1) and Q2=0 and Q3=0 and Q4=0 and Q5=0 and Q6=0 and Q7=0 and Q8=0 and Q9=0] | [Q1=0 or Q1=1 and Q3=0 and Q5=0 and Q6=0 and Q7=0 and Q8=0] |
| Mildly Food Insecure Access | [(Q1=2 or Q1=3 or Q2=1 or Q2=2 or Q2=3 or Q3=1 or Q4=1) and Q5=0 and Q6=0 and Q7=0 and Q8=0 and Q9=0] | [(Q1=2 or Q1=3 or Q3=1] and Q5=0 and Q6=0 and Q7=0 and Q8=0] |
| Moderately Food Insecure Access | [(Q3=2 or Q3=3 or Q4=2 or Q4=3 or Q5=1 or Q5=2 or Q6=1 or Q6=2) and Q7=0 and Q8=0 and Q9=0] | [(Q3=2 or Q3=3 or Q5=2 or Q6=2) and Q7=0 and Q8=0] |
| Severely Food Insecure Access | [Q5=3 or Q6=3 or Q7=1 or Q7=2 or Q7=3 or Q8=1 or Q8=2 or Q8=3 or Q9=1 or Q9=2 or Q9=3] | [Q5=3 or Q6=3 or Q7=1 or Q7=2 or Q7=3 or Q8=1 or Q8=2 or Q8=3] |

- All the shocks variables (increase in the price of food, job loss, theft or destruction of property, crops failed, illness of a family member) come from the shocks module that measure whether the participant’s household economy was affected by any of these events (1 if yes, 0 otherwise).
- Time use: The variable “Hours spent on caring/domestic chores” measures the number of hours the participant dedicates to these activities during a typical day (from Monday to Friday). Similarly, “Spent 8-9 hours of sleep” is calculated from the number of hours the participant sleeps on a typical day. Both variables come from the self-reported Time Allocation module. Furthermore, participants are categorized based on whether they study only, work only, both study and work, or neither. This categorization derives from responses to the Education and Employment modules. In the estimates, 'study and work’ is the reference category.
- The variables “Ever drink alcohol”, “Ever experienced Inter-Personal Violence (IPV)”, and “Ever bullied over the internet” were asked to the participants using the Self-Administered Questionnaires (SAQ) in Peru and Audio Computer-assisted Self-interviews (ACASI) in Ethiopia and India. In Ethiopia, it has been documented that using self-administered survey tools, such as ACASI, rather than face-to-face interviewer-administered interviews, leads to higher disclosure rates of sensitive information (von Russdorf et al., 2024).
- Migration and other variables: migration is defined according to whether the participant has migrated from an urban area to a rural area (or vice versa) between ages 1 and 22. The change in wealth index (WI) measures the difference between the household’s WI at ages 22 and age 1. A positive value means the participant’s WI improved over time.

**Appendix A.2: Descriptive results including Amhara phone survey sample in Ethiopia**


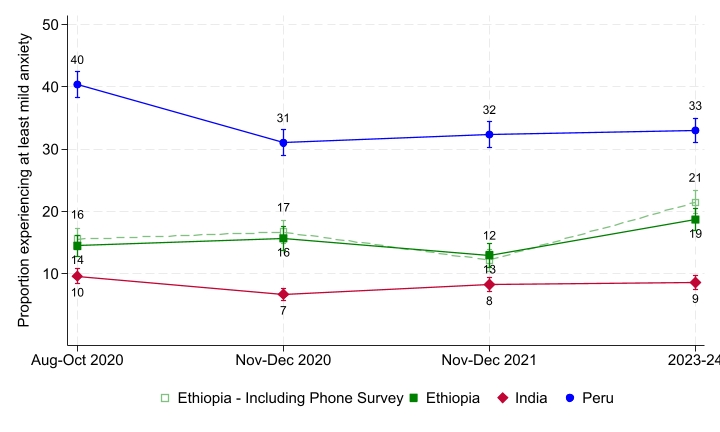


**Figure A.1**. Proportion experiencing at least mild anxiety symptoms, 2020-2024, including participants interviewed via phone survey.

*Notes*: Analysis is performed on a balanced sample of Young Lives respondents that are present in all four rounds. We include the sample in Ethiopia interviewed via phone survey in the dotted line. Vertical bars represent 90% confidence intervals.

**Appendix A.3 Conceptual framework: Life-course predictors of adult mental health**

*Sex*

Previous literature has consistently identified sex and gender as key determinants of mental health. Girls and women tend to exhibit a higher prevalence of “internalizing” disorders, such as depression and anxiety. In contrast, boys and men tend to experience higher rates of “externalizing” disorders, such as aggression, hyperactivity, and risky behaviours (Smith et al., 2018). Potential mechanisms for this disparity include differences in socialisation, help-seeking behaviours, copying strategies, and symptom disclosure.

In many countries, women face additional risk factors that contribute to poorer mental health in adulthood, including increased exposure to domestic and sexual violence and limitations on autonomy in decision-making and reproductive choices (Patel et al., 2006). Furthermore, women frequently bear a disproportionate burden of domestic work compared to men, which can negatively affect their mental health (Carneiro et al., 2023). Evidence from Young Lives data demonstrates that women’s mental health was disproportionately affected during the pandemic, largely due to interrupted education and increased domestic work responsibilities (Ford and Freund, 2022).

*Early-life conditions*

Prior research has linked early-life adversity to poorer mental health outcomes in adulthood. Socioeconomic status, maternal well-being, and early-life health are among the most extensively documented risk factors (Angelini et al., 2020; Fryers and Brugha, 2013). As highlighted by Herba et al. (2016), maternal depression has been associated with worse mental health among their children through multiple channels, including a poorer mother-child relationship, increased exposure to violence in the home, and parental distress that leads to harsher punishments.

Longitudinal evidence from high-income countries also suggests that individuals who grew up in poorer households tend to experience worse mental health in adulthood (Elovainio et al., 2020; Melchior et al., 2007). Key mediating factors identified include persistent socioeconomic vulnerability, family susceptibility to mental and physical illnesses, childhood and adolescent health issues, and exposure to maltreatment. Supporting this, evidence from Ghana by Adhvaryu et al. (2019) shows that a positive income shock during early life significantly reduces the likelihood of severe mental distress in adulthood, with maternal nutrition and increased childhood investments identified as key mechanisms.

The area of residence at birth also potentially influences adult mental health through multiple channels. Being born in a rural area may have long-term negative effects due to higher exposure to rural poverty, child labour, and lower educational attainment, as children in these areas are more likely to work and less likely to attend school (Kruger, 2004; Shen and Hannum, 2023). Conversely, children raised in urban or well-connected areas benefit from greater access to healthcare resources (Peterson et al., 2009) and economic opportunities, both of which are associated with better mental health outcomes. However, urban environments also pose risks, including greater exposure to social inequality, violence, crime, pollution, and limited access to green spaces, all of which have been linked to poorer mental health (Oh et al., 2021; Ventriglio et al., 2021).

*Adolescent experiences*

Adolescence is a transitional period marked by profound changes, during which individuals develop personality traits that become increasingly stable with age and serve as significant predictors for mental health outcomes (Nebhinani and Jain, 2019; Patel et al., 2018). Prior research, largely from high-income countries, highlights that socioemotional skills developed during adolescence influence mental health later in life. For instance, adolescents with higher agency tend to achieve greater socioeconomic status but may experience mental health declines due to unachieved expectations (Hitlin et al., 2015). Self-conscious emotions, such as pride and shame, also play an important role in mental health. Pride, which increases with age, is positively associated with psychological well-being (Orth et al., 2010) and better mental health (Gruber et al., 2011; Nesbitt et al., 2023; Van Doren et al., 2019). Conversely, shame memories during adolescence can increase anxiety and depression in adulthood (Cunha et al., 2012). Agency has been found to be correlated with better mental health for women (Haight et al., 2025; Leight et al., 2022; Yount et al., 2014), but the evidence for the adolescence period is still scarce.

Another key aspect during adolescence is the development of social capital. Positive social relationships during this period can foster better mental health in adulthood. In particular, parent-child relationships characterised by affection and low control have been identified as protective factors for adult mental health (Fryers and Brugha, 2013).

The role of body mass index (BMI) has also been explored. BMI could be related to mental health status for biological reasons. There might also be poor health, social stigma, and body-image associated to obesity that might also play a role (Amin et al., 2020; Steptoe and Frank, 2023). Substantial evidence links obesity with mental health difficulties, particularly depression (Frank et al., 2022; Steptoe and Frank, 2023), however the evidence for young people is less clear (Amin et al., 2020). Furthermore, most of the evidence comes from high-income countries.

Finally, the literature highlights relationships among educational attainment, performance during adolescence, and mental health in adulthood. Poor academic performance and lower standardized test scores in adolescence are associated with adult anxiety, depression, and substance use (Fryers and Brugha, 2013; Koenen et al., 2009; Weckström et al., 2023). Conversely, higher educational achievement is linked to improved psychological resilience and better social and economic circumstances, which can have protective effects on mental health (Brännlund et al., 2017; Veldman et al., 2014).

*Contemporaneous experiences during young adulthood*

Just as high-quality relationships are crucial during adolescence, higher social capital in adulthood, measured through interpersonal trust and social networks, positively correlates with better self-reported mental health (Martínez et al., 2019). In particular, interpersonal and institutional trust serve as protective factors against anxiety and depression. Likewise, research shows that personality traits such as neuroticism and extroversion are significant predictors of adult mental health. Neuroticism (or emotional instability) is positively associated with psychiatric conditions and poorer mental health in adulthood, while extroversion has a protective effect (Fryers and Brugha, 2013; Newton-Howes et al., 2015). In particular, neuroticism is common among adults with depression and anxiety disorders, with rumination and worry serving as mediating mechanisms (Jylhä and Isometsä, 2006; Weinstock and Whisman, 2006; Roelofs et al., 2008). On the other hand, grit has been identified as a predictor of better mental health outcomes (Musumari et al., 2018; Sharma et al., 2021).

Contemporaneous experiences during young adulthood have also been shown to influence mental health. How individuals allocate their time is one predictor of mental health. For instance, psychosocial stressors in the work environment, such as high demands paired with limited decision-making power or high effort with inadequate rewards, can negatively impact mental health (Stansfeld and Candy, 2006). Additionally, individuals with disabilities often face barriers to employment and job insecurity, which exacerbate mental health challenges (Aitken et al., 2018; Honey et al., 2011). The literature also highlights that balancing study, work, and financial hardship is a stressor that can contribute to poorer mental health (Landstedt et al., 2017). Similarly, financial strain is commonly associated with poorer mental health, with contributing factors including insecurity, hopelessness, exposure to unpredictable employment changes, discrimination, risks of violence, and poor physical health (Freund et al., 2025; Patel and Kleinman, 2003).

Food insecurity and poor diet quality are also associated with worse mental health outcomes (Afulani et al., 2020; Alegría et al., 2018). During the COVID-19 pandemic, heightened food insecurity was associated with increased anxiety and depression, particularly among low-income groups (Fang et al., 2021) and individuals in LMICs (Porter et al., 2021). Rural populations also face unique stressors, such as weather-related changes and economic uncertainty, which can contribute to heightened anxiety, depression, and sleep difficulties (Freund, 2023; Kurniyawan et al., 2024; Njeru et al., 2022).

Engaging in risky behaviours, such as alcohol and drug consumption or unprotected sex, is also associated with poorer mental health among young adults (Escobar et al., 2020; Jao et al., 2019). Conversely, positive behaviours, such as regular exercise and healthy sleeping patterns, are correlated with better mental health (Scott et al., 2021).

Exposure to shocks can significantly impact mental health. Negative events, such as the illness or death of a family member or a sudden loss of income, have been shown to affect mental health (Cleland et al., 2016). Similarly, exposure to violence is strongly correlated with worse mental health. For instance, experiencing Intimate Partner Violence (IPV), or violence perpetrated by other family members, negatively impacts mental health (Coker et al., 2002; Golding, 1999; Howard and MacManus, 2020; Meekers et al., 2013). Additionally, exposure to armed conflict has been associated with worse mental health outcomes (Favara et al., 2022). Conversely, positive life events, such as securing a new job or marriage, can have protective effects on mental health (Cleland et al., 2016).

Finally, migration can also play a role in mental health. The effect is ambiguous; it depends on both the reasons for the migration as well as the result of it. Considering the impact of migration on mental health, the literature has found evidence of negative effects associated to the stressful nature of the migration process both to other countries (Bartram, 2011; Bhugra and Jones, 2001) and within one country (Knight and Gunatilaka, 2010). However, other studies have found the impact to be positive (Stillman et al., 2015), indeed the results appear to be strongly sensitive to the characteristics of both the place of origin and the place of destiny. With some exceptions (Knight and Gunatilaka, 2010), we have been unable to identify literature that analyses the impact of internal migration on mental health, but presumably the mechanisms are similar to those observed for internal migration, and the sign of the impact is also likely to be ambiguous.

**Appendix A.4**

**Table A.4: Factors affecting the likelihood of exhibiting anxiety (at least mild, %) – Including participants interviewed via phone survey**

|  | Ethiopia | Ethiopia |
| --- | --- | --- |
|  | (1) | (2) |
| Female | 0.007 | 0.018 |
|  | (0.021) | (0.023) |
| **Early life conditions (Age 1, Round 1)** |  |  |
| Caregivers’ Mental Health Score (Higher=Worse) | 0.003 | 0.002 |
|  | (0.002) | (0.002) |
| Caregiver is literate | -0.050* | -0.045* |
|  | (0.027) | (0.027) |
| Wealth Index | -0.081 | -0.030 |
|  | (0.099) | (0.103) |
| Urban | 0.076** | 0.081** |
|  | (0.037) | (0.038) |
| **Adolescence factors and experiences (Age 15, Round 5)** |  |  |
| Parent-child relationship |  | -0.012 |
|  |  | (0.021) |
| Pride Index (z-score) |  | -0.058*** |
|  |  | (0.020) |
| Agency Index (z-score) |  | 0.054*** |
|  |  | (0.020) |
| Math Test (% Correct) |  | 0.001 |
|  |  | (0.001) |
| Study Hours (Last Week) |  | -0.028*** |
|  |  | (0.008) |
| Enrolled in School |  | 0.108* |
|  |  | (0.064) |
| BMI (z-score) |  | -0.002 |
|  |  | (0.008) |
| Change in BMI (age 5-15) |  | -0.001 |
|  |  | (0.008) |
| Constant | 0.103*** | 0.184 |
|  | (0.025) | (0.129) |
| Observations | 1,352 | 1,352 |
| R-squared | 0.101 | 0.125 |
| Round 1 Ethnicity FE | Yes | Yes |
| Round 7 Region FE | No | No |
| *Notes*: Results of Column (2) control for missing values for the mathematics score in Round 5. Robust standard errors are reported in parentheses. *** p<0.01, ** p<0.05, * p<0.1 | | |

**Table A.5: Test of Equality of Coefficients Across Country Samples for Anxiety**

|  | Model specification (1) | | Model specification (2) | | Model specification (3) | |  |  |  |  |  |  |  |  |  |  |  |  |  |
| --- | --- | --- | --- | --- | --- | --- | --- | --- | --- | --- | --- | --- | --- | --- | --- | --- | --- | --- | --- |
|  | chi2 | p | chi2 | p | chi2 | p |  |  |  |  |  |  |  |  |  |  |  |  |  |
|  |  |  |  |  |  |  |  |  |  |  |  |  |  |  |  |  |  |  |  |
| Female | 44 | 0.000 | 34 | 0.000 | 8 | 0.017 |  |  |  |  |  |  |  |  |  |  |  |  |  |
|  |  |  |  |  |  |  |  |  |  |  |  |  |  |  |  |  |  |  |  |
| **Early life conditions (Age 1, Round 1)** |  |  |  |  |  |  |  |  |  |  |  |  |  |  |  |  |  |  |  |
| Caregivers’ Mental Health Score (Higher=Worse) | 11 | 0.004 | 11 | 0.005 | 8 | 0.023 |  |  |  |  |  |  |  |  |  |  |  |  |  |
| Caregiver is literate | 3 | 0.230 | 3 | 0.230 | 1 | 0.483 |  |  |  |  |  |  |  |  |  |  |  |  |  |
| Wealth Index | 8 | 0.021 | 7 | 0.031 | 3 | 0.273 |  |  |  |  |  |  |  |  |  |  |  |  |  |
| Urban | 6 | 0.059 | 7 | 0.032 | 9 | 0.010 |  |  |  |  |  |  |  |  |  |  |  |  |  |
| **Adolescence factors and experiences (Age 15, Round 5)** | | | | | | | | | | | | | | | |  |  |  |  |
| Parent-child relationship |  |  | 4 | 0.131 | 4 | 0.131 |  |  |  |  |  |  |  |  |  |  |  |  |  |
| Pride Index (z-score) |  |  | 6 | 0.039 | 2 | 0.328 |  |  |  |  |  |  |  |  |  |  |  |  |  |
| Agency index (z-score) |  |  | 2 | 0.321 | 3 | 0.267 |  |  |  |  |  |  |  |  |  |  |  |  |  |
| Math Test (% Correct) |  |  | 4 | 0.133 | 1 | 0.622 |  |  |  |  |  |  |  |  |  |  |  |  |  |
| Study Hours (Last Week) |  |  | 8 | 0.023 | 7 | 0.033 |  |  |  |  |  |  |  |  |  |  |  |  |  |
| Enrolled in school |  |  | 2 | 0.352 | 3 | 0.260 |  |  |  |  |  |  |  |  |  |  |  |  |  |
| BMI |  |  | 1 | 0.707 | 0 | 0.919 |  |  |  |  |  |  |  |  |  |  |  |  |  |
| Change in BMI (Round 5 vs Round 2) |  |  | 0 | 0.932 | 1 | 0.752 |  |  |  |  |  |  |  |  |  |  |  |  |  |
| **Early adulthood experiences (Age 22, Round 7)** | | | | | | | | | | | | | | |  |  |  |  |  |
| High interpersonal trust |  |  |  |  | 4 | 0.133 |  |  |  |  |  |  |  |  |  |  |  |  |  |
| Big 5 Neuroticism (z-score) |  |  |  |  | 61 | 0.000 |  |  |  |  |  |  |  |  |  |  |  |  |  |
| Grit Index (z-score) |  |  |  |  | 6 | 0.061 |  |  |  |  |  |  |  |  |  |  |  |  |  |
| Food insecure |  |  |  |  | 8 | 0.021 |  |  |  |  |  |  |  |  |  |  |  |  |  |
| Increase in the price of food I buy |  |  |  |  | 2 | 0.355 |  |  |  |  |  |  |  |  |  |  |  |  |  |
| Job loss/source of income/family enterprise |  |  |  |  | 2 | 0.412 |  |  |  |  |  |  |  |  |  |  |  |  |  |
| Theft or destruction of property |  |  |  |  | 2 | 0.353 |  |  |  |  |  |  |  |  |  |  |  |  |  |
| Crops failed |  |  |  |  | 11 | 0.004 |  |  |  |  |  |  |  |  |  |  |  |  |  |
| Illness of a family member |  |  |  |  | 1 | 0.509 |  |  |  |  |  |  |  |  |  |  |  |  |  |
| Hours spent on caring / domestic chores past week |  |  |  |  | 2 | 0.297 |  |  |  |  |  |  |  |  |  |  |  |  |  |
| Spent 8-9 hours sleeping past week (Round 7) |  |  |  |  | 1 | 0.710 |  |  |  |  |  |  |  |  |  |  |  |  |  |
| Disability affecting work / capacity take care of yourself |  |  |  |  | 0 | 0.940 |  |  |  |  |  |  |  |  |  |  |  |  |  |
| Physical attacked due to conflict (includes attempt) |  |  |  |  | NA | NA |  |  |  |  |  |  |  |  |  |  |  |  |  |
| Ever experienced IPV |  |  |  |  | 12 | 0.003 |  |  |  |  |  |  |  |  |  |  |  |  |  |
| Ever been bullied over the internet |  |  |  |  | 1 | 0.531 |  |  |  |  |  |  |  |  |  |  |  |  |  |
| Ever married or cohabitating |  |  |  |  | 1 | 0.552 |  |  |  |  |  |  |  |  |  |  |  |  |  |
| Underage marriage/cohabitation or teen parenthood |  |  |  |  | 7 | 0.036 |  |  |  |  |  |  |  |  |  |  |  |  |  |
| Ever drunk alcohol |  |  |  |  | 2 | 0.328 |  |  |  |  |  |  |  |  |  |  |  |  |  |
| Studying only |  |  |  |  | 5 | 0.094 |  |  |  |  |  |  |  |  |  |  |  |  |  |
| Working only |  |  |  |  | 3 | 0.203 |  |  |  |  |  |  |  |  |  |  |  |  |  |
| Neither working nor studying |  |  |  |  | 0 | 0.925 |  |  |  |  |  |  |  |  |  |  |  |  |  |
| Highest education level: Primary education |  |  |  |  | 2 | 0.338 |  |  |  |  |  |  |  |  |  |  |  |  |  |
| Highest education level: Secondary education |  |  |  |  | 4 | 0.113 |  |  |  |  |  |  |  |  |  |  |  |  |  |
| Highest education level: Higher education |  |  |  |  | 4 | 0.119 |  |  |  |  |  |  |  |  |  |  |  |  |  |
| Change in wealth index (Round 7 vs Round 1) |  |  |  |  | 1 | 0.603 |  |  |  |  |  |  |  |  |  |  |  |  |  |

The null hypothesis is that the coefficients reported in Table 1 are the same across countries. Model specification (1) corresponds to the model that takes into account early-life conditions, (2) includes early life conditions as well as adolescence factors and experiences, and (3) includes all those factors as well as early adulthood experiences. Results obtained using the suest command in STATA, for the common set of variables across countries.

**Table A.6: Factors affecting the likelihood of exhibiting depression (at least mild, %)**

|  | **Ethiopia** | | | **India** | | | **Peru** | | |
| --- | --- | --- | --- | --- | --- | --- | --- | --- | --- |
|  | (1) | (2) | (3) | (1) | (2) | (3) | (1) | (2) | (3) |
| Female | 0.034* | 0.021 | 0.012 | 0.017 | 0.021 | 0.030 | 0.174*** | 0.158*** | 0.062** |
|  | (0.020) | (0.021) | (0.024) | (0.014) | (0.015) | (0.020) | (0.022) | (0.024) | (0.026) |
| **Early life conditions (Age 1, Round 1)** |  |  |  |  |  |  |  |  |  |
|  |  |  |  |  |  |  |  |  |  |
| Caregivers’ Mental Health Score (Higher=Worse) | 0.002 | 0.001 | -0.001 | 0.002 | 0.002 | -0.000 | 0.008*** | 0.006** | 0.001 |
|  | (0.002) | (0.002) | (0.002) | (0.002) | (0.002) | (0.002) | (0.003) | (0.003) | (0.003) |
| Caregiver is literate | -0.058** | -0.057** | -0.051** | -0.016 | -0.011 | -0.014 | -0.000 | -0.001 | -0.009 |
|  | (0.024) | (0.024) | (0.024) | (0.016) | (0.016) | (0.017) | (0.032) | (0.031) | (0.029) |
| Wealth Index | 0.130 | 0.145 | 0.272** | -0.063 | -0.040 | -0.046 | 0.220*** | 0.207*** | 0.143 |
|  | (0.088) | (0.093) | (0.115) | (0.050) | (0.052) | (0.086) | (0.062) | (0.065) | (0.093) |
| Urban | -0.006 | -0.006 | -0.030 | 0.020 | 0.020 | 0.008 | 0.015 | 0.003 | -0.003 |
|  | (0.033) | (0.034) | (0.036) | (0.021) | (0.022) | (0.022) | (0.034) | (0.034) | (0.037) |
| **Adolescence factors and experiences (Age 15, Round 5)** |  |  |  |  |  |  |  |  |  |
| Parent-child relationship |  | -0.007 | 0.004 |  | -0.017 | -0.008 |  | -0.078*** | -0.062*** |
|  |  | (0.020) | (0.019) |  | (0.012) | (0.012) |  | (0.020) | (0.018) |
| Pride Index (z-score) |  | -0.026 | -0.011 |  | -0.001 | -0.007 |  | -0.038* | -0.016 |
|  |  | (0.018) | (0.017) |  | (0.011) | (0.011) |  | (0.021) | (0.019) |
| Agency Index (z-score) |  | -0.003 | -0.010 |  | 0.008 | 0.002 |  | 0.057** | 0.061*** |
|  |  | (0.020) | (0.019) |  | (0.014) | (0.014) |  | (0.023) | (0.021) |
| Math Test (% Correct) |  | 0.000 | 0.001 |  | -0.001** | 0.000 |  | 0.000 | 0.001 |
|  |  | (0.001) | (0.001) |  | (0.000) | (0.000) |  | (0.001) | (0.001) |
| Study Hours (Last Week) |  | -0.006 | -0.003 |  | 0.001 | 0.001 |  | -0.001 | 0.000 |
|  |  | (0.008) | (0.007) |  | (0.005) | (0.005) |  | (0.006) | (0.006) |
| Enrolled in School |  | 0.086 | 0.046 |  | -0.003 | 0.017 |  | 0.030 | 0.033 |
|  |  | (0.064) | (0.062) |  | (0.062) | (0.059) |  | (0.081) | (0.078) |
| BMI (z-score) |  | -0.004 | 0.001 |  | 0.003 | 0.003 |  | -0.001 | -0.002 |
|  |  | (0.007) | (0.007) |  | (0.006) | (0.005) |  | (0.007) | (0.006) |
| Change in BMI (age 5-15) |  | 0.012* | 0.006 |  | -0.006 | -0.006 |  | 0.001 | 0.005 |
|  |  | (0.006) | (0.006) |  | (0.006) | (0.005) |  | (0.008) | (0.008) |
| **Early adulthood experiences (Age 22, Round 7)** |  |  |  |  |  |  |  |  |  |
| High interpersonal trust |  |  | -0.043 |  |  | -0.001 |  |  | 0.023 |
|  |  |  | (0.028) |  |  | (0.017) |  |  | (0.041) |
| Big 5 Neuroticism (z-score) |  |  | 0.131*** |  |  | 0.082*** |  |  | 0.214*** |
|  |  |  | (0.022) |  |  | (0.016) |  |  | (0.021) |
| Grit Index (z-score) |  |  | -0.004 |  |  | -0.042** |  |  | -0.122*** |
|  |  |  | (0.024) |  |  | (0.017) |  |  | (0.027) |
| Food insecure |  |  | 0.066*** |  |  | -0.010 |  |  | 0.084*** |
|  |  |  | (0.021) |  |  | (0.019) |  |  | (0.022) |
| Increase in the price of food I buy |  |  | 0.053*** |  |  | 0.026* |  |  | 0.012 |
|  |  |  | (0.020) |  |  | (0.015) |  |  | (0.036) |
| Job loss/source of income/family enterprise |  |  | 0.051* |  |  | 0.033 |  |  | 0.049** |
|  |  |  | (0.029) |  |  | (0.032) |  |  | (0.022) |
| Theft or destruction of property |  |  | 0.092*** |  |  | -0.008 |  |  | 0.013 |
|  |  |  | (0.032) |  |  | (0.036) |  |  | (0.029) |
| Crops failed |  |  | 0.100** |  |  | 0.031* |  |  | 0.063 |
|  |  |  | (0.045) |  |  | (0.017) |  |  | (0.041) |
| Illness of a family member |  |  | 0.064*** |  |  | 0.034** |  |  | 0.033 |
|  |  |  | (0.023) |  |  | (0.016) |  |  | (0.022) |
| Hours spent on caring / domestic chores past week |  |  | -0.002 |  |  | 0.002 |  |  | 0.001 |
|  |  |  | (0.004) |  |  | (0.004) |  |  | (0.005) |
| Spent 8-9 hours sleeping past week |  |  | -0.021 |  |  | -0.029* |  |  | -0.010 |
|  |  |  | (0.021) |  |  | (0.017) |  |  | (0.021) |
| Disability affecting work / capacity take care of yourself |  |  | 0.067 |  |  | 0.015 |  |  | 0.297*** |
|  |  |  | (0.079) |  |  | (0.208) |  |  | (0.066) |
| Ever experienced IPV |  |  | 0.063* |  |  | -0.004 |  |  | 0.131*** |
|  |  |  | (0.035) |  |  | (0.028) |  |  | (0.027) |
| Ever been bullied over the internet |  |  | 0.079* |  |  | 0.029 |  |  | 0.091** |
|  |  |  | (0.041) |  |  | (0.039) |  |  | (0.044) |
| Physical attacked due to conflict (includes attempt) |  |  | 0.042* |  |  |  |  |  |  |
|  |  |  | (0.023) |  |  |  |  |  |  |
| Ever married or cohabitating |  |  | 0.005 |  |  | -0.024 |  |  | -0.076*** |
|  |  |  | (0.043) |  |  | (0.028) |  |  | (0.027) |
| Underage marriage/cohabitation or teen parenthood |  |  | -0.104** |  |  | 0.052* |  |  | -0.015 |
|  |  |  | (0.049) |  |  | (0.030) |  |  | (0.037) |
| Ever drunk alcohol |  |  | 0.006 |  |  | 0.067** |  |  | 0.048* |
|  |  |  | (0.023) |  |  | (0.026) |  |  | (0.028) |
| Studying only |  |  | -0.021 |  |  | -0.058** |  |  | 0.004 |
|  |  |  | (0.033) |  |  | (0.026) |  |  | (0.035) |
| Working only |  |  | -0.009 |  |  | -0.015 |  |  | -0.053** |
|  |  |  | (0.029) |  |  | (0.026) |  |  | (0.027) |
| Neither working nor studying |  |  | 0.041 |  |  | -0.053* |  |  | -0.055 |
|  |  |  | (0.042) |  |  | (0.031) |  |  | (0.057) |
| Highest education level: Primary education |  |  | -0.031 |  |  | -0.008 |  |  | -0.039 |
|  |  |  | (0.031) |  |  | (0.043) |  |  | (0.118) |
| Highest education level: Secondary education |  |  | -0.010 |  |  | -0.026 |  |  | 0.023 |
|  |  |  | (0.030) |  |  | (0.038) |  |  | (0.122) |
| Highest education level: Higher education |  |  | -0.048 |  |  | 0.002 |  |  | 0.012 |
|  |  |  | (0.046) |  |  | (0.040) |  |  | (0.125) |
| Change in Wealth Index (age 1-22) |  |  | 0.152* |  |  | -0.037 |  |  | -0.039 |
|  |  |  | (0.091) |  |  | (0.079) |  |  | (0.088) |
| Migrated (age 1-22) |  |  |  |  |  | -0.020 |  |  | 0.019 |
|  |  |  |  |  |  | (0.021) |  |  | (0.033) |
| Constant | 0.086*** | 0.091 | -0.164 | 0.050* | 0.033 | 0.014 | 0.060 | 0.081 | 0.004 |
|  | (0.025) | (0.116) | (0.138) | (0.027) | (0.088) | (0.115) | (0.037) | (0.137) | (0.172) |
| Observations | 1,231 | 1,231 | 1,231 | 1,716 | 1,716 | 1,716 | 1,558 | 1,558 | 1,558 |
| R-squared | 0.060 | 0.069 | 0.213 | 0.023 | 0.027 | 0.105 | 0.055 | 0.074 | 0.276 |
| Round 1 Ethnicity FE | Yes | Yes | Yes | Yes | Yes | Yes | Yes | Yes | Yes |
| Round 7 Region FE | No | No | Yes | No | No | Yes | No | No | Yes |
| *Notes*: Results of columns (2) and (3) control for missing values for the mathematics score in Round 5. Results in columns (3) control for missing responses on reporting IPV, bullying online, physical attacks due to conflict and drinking alcohol. Robust standard errors are reported in parentheses. *** p<0.01, ** p<0.05, * p<0.1 | | | | | | | | | |

**Table A.7: Test of Equality of Coefficients Across Country Samples for Depression**

|  | Model specification (1) | | Model specification (2) | | Model specification (3) | |
| --- | --- | --- | --- | --- | --- | --- |
|  | chi2 | p | chi2 | p | chi2 | p |
| Female | 36 | 0.000 | 27 | 0.000 | 2 | 0.365 |
| **Early life conditions (Age 1, Round 1)** |  |  |  |  |  |  |
| Caregivers’ Mental Health Score (Higher=Worse) | 3 | 0.180 | 3 | 0.276 | 0 | 0.800 |
| Caregiver is literate | 3 | 0.230 | 3 | 0.211 | 2 | 0.376 |
| Wealth Index | 13 | 0.001 | 10 | 0.008 | 6 | 0.062 |
| Urban | 0 | 0.799 | 0 | 0.792 | 1 | 0.653 |
| **Adolescence factors and experiences (Age 15, Round 5)** | |  |  |  |  |  |
| Parent-child relationship |  |  | 9 | 0.013 | 9 | 0.013 |
| Pride Index (z-score) |  |  | 3 | 0.218 | 0 | 0.922 |
| Agency index (z-score) |  |  | 4 | 0.112 | 7 | 0.024 |
| Math Test (% Correct) |  |  | 2 | 0.354 | 1 | 0.576 |
| Study Hours (Last Week) |  |  | 1 | 0.753 | 0 | 0.851 |
| Enrolled in school |  |  | 1 | 0.597 | 0 | 0.940 |
| BMI |  |  | 1 | 0.680 | 0 | 0.798 |
| Change in BMI (Round 5 vs Round 2) |  |  | 4 | 0.121 | 3 | 0.237 |
| **Early adulthood experiences (Age 22, Round 7)** | |  |  |  |  |  |
| High interpersonal trust |  |  |  |  | 2 | 0.295 |
| Big 5 Neuroticism (z-score) |  |  |  |  | 27 | 0.000 |
| Grit Index (z-score) |  |  |  |  | 11 | 0.004 |
| Food insecure |  |  |  |  | 13 | 0.001 |
| Increase in the price of food I buy |  |  |  |  | 2 | 0.433 |
| Job loss/source of income/family enterprise |  |  |  |  | 0 | 0.892 |
| Theft or destruction of property |  |  |  |  | 5 | 0.066 |
| Crops failed |  |  |  |  | 2 | 0.296 |
| Illness of a family member |  |  |  |  | 1 | 0.495 |
| Hours spent on caring / domestic chores past week |  |  |  |  | 1 | 0.724 |
| Spent 8-9 hours sleeping past week (Round 7) |  |  |  |  | 1 | 0.749 |
| Disability affecting work / capacity take care of yourself |  |  |  |  | 6 | 0.050 |
| Physical attacked due to conflict (includes attempt) |  |  |  |  | NA | NA |
| Ever experienced IPV |  |  |  |  | 13 | 0.002 |
| Ever been bullied over the internet |  |  |  |  | 1 | 0.500 |
| Ever married or cohabitating |  |  |  |  | 3 | 0.196 |
| Underage marriage/cohabitation or teen parenthood |  |  |  |  | 8 | 0.020 |
| Ever drunk alcohol |  |  |  |  | 3 | 0.187 |
| Studying only |  |  |  |  | 2 | 0.325 |
| Working only |  |  |  |  | 2 | 0.449 |
| Neither working nor studying |  |  |  |  | 4 | 0.158 |
| Highest education level: Primary education |  |  |  |  | 0 | 0.893 |
| Highest education level: Secondary education |  |  |  |  | 0 | 0.898 |
| Highest education level: Higher education |  |  |  |  | 1 | 0.689 |
| Change in wealth index (Round 7 vs Round 1) |  |  |  |  | 3 | 0.203 |

The null hypothesis is that the coefficients reported in Table A.6 are the same across countries. Model specification (1) corresponds to the model that takes into account early-life conditions, (2) includes early life conditions as well as adolescence factors and experiences, and (3) includes all those factors as well as early adulthood experiences. Results obtained using the suest command in STATA, for the common set of variables across countries.

**Table A.8: Value-added regression on depression prevalence (at least mild, %)**

|  |  |  |  | |  |
| --- | --- | --- | --- | --- | --- |
|  | Ethiopia | India | Peru | |  |
|  | (1) | (2) | (3) | |  |
| At least mild depression (2020) | 0.027 | 0.117*** | | 0.150*** | |
|  | (0.031) | (0.033) | | (0.027) | |
| Female | 0.023 | 0.028 | | 0.089*** | |
|  | (0.024) | (0.020) | | (0.026) | |
| **Early adulthood experiences (Age 22, Round 7)** |  |  | |  | |
| High interpersonal trust | -0.049* | 0.004 | | 0.023 | |
|  | (0.028) | (0.016) | | (0.044) | |
| Big 5 Neuroticism (z-score) | 0.126*** | 0.079*** | | 0.181*** | |
|  | (0.022) | (0.016) | | (0.022) | |
| Grit Index (z-score) | 0.004 | -0.048*** | | -0.129*** | |
|  | (0.024) | (0.017) | | (0.029) | |
| Food insecure | 0.054*** | -0.008 | | 0.058** | |
|  | (0.020) | (0.017) | | (0.023) | |
| Increase in the price of food I buy | 0.063*** | 0.029* | | -0.006 | |
|  | (0.020) | (0.015) | | (0.037) | |
| Job loss/source of income/family enterprise | 0.052* | 0.021 | | 0.061*** | |
|  | (0.028) | (0.031) | | (0.023) | |
| Theft or destruction of property | 0.097*** | -0.011 | | 0.030 | |
|  | (0.033) | (0.034) | | (0.031) | |
| Crops failed | 0.089** | 0.041** | | 0.029 | |
|  | (0.045) | (0.017) | | (0.042) | |
| Illness of a family member | 0.058** | 0.036** | | 0.019 | |
|  | (0.023) | (0.015) | | (0.023) | |
| Hours spent on caring/domestic chores past week | -0.003 | 0.002 | | 0.002 | |
|  | (0.004) | (0.004) | | (0.005) | |
| Spent 8-9 hours sleeping past week | -0.028 | -0.028* | | -0.019 | |
|  | (0.021) | (0.016) | | (0.022) | |
| Disability affecting work / capacity take care of yourself | 0.075 | 0.058 | | 0.296*** | |
|  | (0.080) | (0.211) | | (0.071) | |
| Ever experienced IPV | 0.059* | -0.006 | | 0.111*** | |
|  | (0.034) | (0.028) | | (0.028) | |
| Ever been bullied over the internet | 0.067 | 0.034 | | 0.076* | |
|  | (0.041) | (0.038) | | (0.046) | |
| Physical attacked due to conflict (includes attempt) | 0.037 |  | |  | |
|  | (0.023) |  | |  | |
| Ever married or cohabitating | 0.017 | -0.031 | | -0.083*** | |
|  | (0.041) | (0.027) | | (0.029) | |
| Underage marriage/cohabitation or teen parenthood | -0.124*** | 0.050* | | -0.029 | |
|  | (0.046) | (0.030) | | (0.041) | |
| Ever drunk alcohol | 0.013 | 0.066** | | 0.087*** | |
|  | (0.024) | (0.026) | | (0.028) | |
| Studying only | -0.013 | -0.057** | | 0.006 | |
|  | (0.034) | (0.025) | | (0.035) | |
| Working only | -0.005 | -0.009 | | -0.070*** | |
|  | (0.029) | (0.024) | | (0.027) | |
| Neither working nor studying | 0.051 | -0.047 | | -0.101* | |
|  | (0.042) | (0.030) | | (0.060) | |
| Highest education level: Primary education | -0.017 | -0.020 | | 0.064 | |
|  | (0.030) | (0.043) | | (0.167) | |
| Highest education level: Secondary education | 0.010 | -0.021 | | 0.097 | |
|  | (0.027) | (0.037) | | (0.164) | |
| Highest education level: Higher education | -0.033 | -0.010 | | 0.085 | |
|  | (0.042) | (0.038) | | (0.166) | |
| Constant | -0.039 | 0.077 | | 0.006 | |
|  | (0.051) | (0.049) | | (0.170) | |
| Observations | 1,183 | 1,710 | | 1,376 | |
| R-squared | 0.208 | 0.103 | | 0.280 | |
| Round 1 Ethnicity FE | No | No | No | |  |
| Round 7 Region FE | Yes | Yes | Yes | |  |

**Table A.9: Factors affecting the likelihood of exhibiting anxiety (at least mild, %) by sex**

|  | **Ethiopia** | | **India** | | **Peru** | |
| --- | --- | --- | --- | --- | --- | --- |
|  | Male | Female | Male | Female | Male | Female |
|  | (1) | (2) | (3) | (4) | (5) | (6) |
| **Early life conditions (Age 1, Round 1)** |  |  |  |  |  |  |
| Caregivers’ Mental Health Score (Higher=Worse) | 0.000 | -0.000 | -0.004 | -0.005* | 0.007* | 0.001 |
|  | (0.003) | (0.003) | (0.002) | (0.003) | (0.004) | (0.004) |
| Caregiver is literate | -0.052 | 0.007 | 0.002 | -0.050** | -0.006 | 0.030 |
|  | (0.038) | (0.037) | (0.023) | (0.024) | (0.040) | (0.044) |
| Wealth Index | 0.105 | 0.077 | 0.094 | -0.097 | 0.292** | 0.097 |
|  | (0.166) | (0.188) | (0.105) | (0.132) | (0.126) | (0.141) |
| Urban | 0.156*** | 0.068 | -0.029 | -0.017 | 0.070 | 0.089 |
|  | (0.055) | (0.058) | (0.028) | (0.035) | (0.047) | (0.060) |
| **Adolescence factors and experiences (Age 15, Round 5)** |  |  |  |  |  |  |
| Parent-child relationship | 0.044 | 0.002 | 0.003 | -0.034* | -0.035 | -0.029 |
|  | (0.028) | (0.028) | (0.014) | (0.019) | (0.026) | (0.024) |
| Pride Index (z-score) | -0.041 | -0.031 | -0.005 | -0.003 | -0.009 | 0.000 |
|  | (0.026) | (0.027) | (0.014) | (0.015) | (0.030) | (0.026) |
| Math Test (% Correct) | 0.032 | 0.023 | -0.004 | -0.007 | 0.051* | -0.017 |
|  | (0.030) | (0.029) | (0.016) | (0.020) | (0.030) | (0.031) |
| Study Hours (Last Week) | 0.001 | 0.001 | -0.001* | 0.001 | -0.001 | 0.001 |
|  | (0.001) | (0.001) | (0.001) | (0.001) | (0.001) | (0.001) |
| Enrolled in School | 0.070 | 0.038 | 0.012 | 0.072 | -0.070 | -0.102 |
|  | (0.080) | (0.096) | (0.056) | (0.085) | (0.101) | (0.126) |
| BMI (z-score) | -0.004 | 0.009 | 0.008 | 0.003 | 0.006 | -0.002 |
|  | (0.011) | (0.011) | (0.006) | (0.008) | (0.010) | (0.007) |
| Change in BMI (age 5-15) | -0.007 | -0.006 | -0.011 | 0.004 | -0.003 | 0.006 |
|  | (0.011) | (0.011) | (0.007) | (0.008) | (0.011) | (0.008) |
| **Early adulthood experiences (Age 22, Round 7)** |  |  |  |  |  |  |
| High interpersonal trust | -0.066* | -0.082* | 0.006 | -0.053* | 0.014 | 0.028 |
|  | (0.040) | (0.049) | (0.018) | (0.028) | (0.050) | (0.078) |
| Big 5 Neuroticism (z-score) | 0.157*** | 0.160*** | 0.069*** | 0.077*** | 0.225*** | 0.297*** |
|  | (0.035) | (0.030) | (0.020) | (0.022) | (0.030) | (0.029) |
| Grit Index (z-score) | 0.059 | -0.013 | -0.044** | -0.023 | 0.002 | -0.038 |
|  | (0.038) | (0.039) | (0.021) | (0.025) | (0.037) | (0.042) |
| Food insecure | 0.083** | 0.048 | -0.045* | 0.035 | 0.029 | 0.105*** |
|  | (0.035) | (0.035) | (0.027) | (0.024) | (0.032) | (0.036) |
| Increase in the price of food I buy | 0.013 | -0.011 | -0.026 | 0.024 | 0.044 | 0.054 |
|  | (0.031) | (0.041) | (0.021) | (0.023) | (0.042) | (0.058) |
| Job loss/source of income/family enterprise | 0.111*** | 0.011 | 0.016 | 0.092* | 0.051* | -0.009 |
|  | (0.040) | (0.048) | (0.036) | (0.055) | (0.031) | (0.033) |
| Theft or destruction of property | 0.073 | 0.071 | 0.115** | 0.104 | 0.067* | 0.013 |
|  | (0.047) | (0.046) | (0.052) | (0.075) | (0.038) | (0.045) |
| Crops failed | 0.097 | 0.162** | 0.008 | -0.001 | 0.134** | 0.087 |
|  | (0.060) | (0.076) | (0.019) | (0.028) | (0.062) | (0.059) |
| Illness of a family member | 0.056 | 0.093*** | 0.047*** | 0.036 | 0.011 | 0.104*** |
|  | (0.037) | (0.035) | (0.017) | (0.025) | (0.030) | (0.034) |
| Hours spent on caring/domestic chores past week | 0.006 | 0.012* | 0.010 | -0.004 | 0.001 | -0.002 |
|  | (0.007) | (0.006) | (0.007) | (0.005) | (0.009) | (0.006) |
| Spent 8-9 hours sleeping past week | 0.000 | -0.025 | -0.032 | 0.011 | -0.044 | -0.017 |
|  | (0.033) | (0.033) | (0.020) | (0.024) | (0.028) | (0.032) |
| Disability affecting work / capacity take care of yourself | 0.162* | 0.003 | 0.218 | 0.177 | 0.215** | -0.061 |
|  | (0.091) | (0.136) | (0.270) | (0.424) | (0.105) | (0.139) |
| Ever experienced IPV | 0.052 | 0.074 | 0.078* | 0.033 | 0.204*** | 0.163*** |
|  | (0.049) | (0.052) | (0.047) | (0.042) | (0.040) | (0.038) |
| Ever been bullied over the internet | -0.001 | 0.187** | 0.075 | -0.020 | -0.060 | 0.022 |
|  | (0.050) | (0.088) | (0.050) | (0.074) | (0.068) | (0.058) |
| Physical attacked due to conflict (includes attempt) | 0.045 | 0.018 |  |  |  |  |
|  | (0.034) | (0.043) |  |  |  |  |
| Ever married or cohabitating | 0.042 | -0.013 | -0.058 | -0.022 | 0.064 | -0.070 |
|  | (0.070) | (0.052) | (0.055) | (0.034) | (0.045) | (0.044) |
| Underage marriage/cohabitation or teen parenthood | -0.082 | -0.075 | 0.010 | 0.101*** | 0.041 | 0.086* |
|  | (0.149) | (0.062) | (0.070) | (0.033) | (0.066) | (0.049) |
| Ever drunk alcohol | 0.002 | -0.046 | 0.027 | 0.206* | -0.018 | 0.023 |
|  | (0.035) | (0.040) | (0.023) | (0.112) | (0.049) | (0.041) |
| Studying only | -0.010 | -0.032 | 0.010 | -0.036 | 0.103** | 0.089* |
|  | (0.053) | (0.051) | (0.021) | (0.063) | (0.052) | (0.051) |
| Working only | -0.025 | -0.041 | 0.034 | -0.031 | -0.014 | 0.066 |
|  | (0.037) | (0.052) | (0.025) | (0.062) | (0.035) | (0.042) |
| Neither working nor studying | 0.123* | -0.081 | 0.054 | -0.053 | 0.085 | 0.037 |
|  | (0.073) | (0.063) | (0.038) | (0.062) | (0.152) | (0.074) |
| Highest education level: Primary education | -0.020 | -0.066 | -0.036 | -0.051 | 0.216* | 0.119 |
|  | (0.043) | (0.050) | (0.052) | (0.065) | (0.118) | (0.232) |
| Highest education level: Secondary education | 0.003 | -0.077* | -0.045 | -0.021 | 0.351*** | 0.164 |
|  | (0.047) | (0.046) | (0.054) | (0.055) | (0.123) | (0.240) |
| Highest education level: Higher education | -0.067 | 0.064 | -0.042 | -0.053 | 0.265** | 0.176 |
|  | (0.084) | (0.070) | (0.054) | (0.061) | (0.127) | (0.243) |
| Change in Wealth Index (age 1-22) | 0.198 | 0.078 | 0.074 | -0.077 | 0.088 | -0.013 |
|  | (0.130) | (0.148) | (0.088) | (0.124) | (0.126) | (0.141) |
| Migrated (age 1-22) |  |  | -0.025 | -0.021 | 0.057 | 0.108** |
|  |  |  | (0.033) | (0.035) | (0.042) | (0.055) |
| Constant | -0.072 | -0.023 | -0.076 | 0.024 | -0.424* | -0.069 |
|  | (0.206) | (0.221) | (0.127) | (0.173) | (0.220) | (0.284) |
| Observations | 619 | 612 | 935 | 781 | 764 | 794 |
| R-squared | 0.312 | 0.283 | 0.134 | 0.181 | 0.257 | 0.295 |
| Round 1 Ethnicity FE | Yes | Yes | Yes | Yes | Yes | Yes |
| Round 7 Region FE | Yes | Yes | Yes | Yes | Yes | Yes |
| Notes: Results control for missing values for the mathematics score in Round 5. We also control for missing responses on reporting IPV, bullying online, physical attacks due to conflict and drinking alcohol. Robust standard errors are reported in parentheses. *** p<0.01, ** p<0.05, * p<0.1 | | | | | | |

**Table A.10: Factors affecting the likelihood of exhibiting depression (at least mild, %) by sex**

|  | **Ethiopia** | | **India** | | **Peru** | |
| --- | --- | --- | --- | --- | --- | --- |
|  | Male | Female | Male | Female | Male | Female |
|  | (1) | (2) | (3) | (4) | (5) | (6) |
| **Early life conditions (Age 1, Round 1)** |  |  |  |  |  |  |
| Caregivers’ Mental Health Score (Higher=Worse) | -0.001 | -0.001 | 0.001 | -0.002 | 0.004 | -0.002 |
|  | (0.003) | (0.003) | (0.002) | (0.003) | (0.004) | (0.004) |
| Caregiver is literate | -0.053* | -0.034 | -0.021 | -0.020 | -0.009 | -0.003 |
|  | (0.031) | (0.036) | (0.024) | (0.024) | (0.039) | (0.043) |
| Wealth Index | 0.250* | 0.260 | 0.069 | -0.199 | 0.208* | 0.094 |
|  | (0.145) | (0.180) | (0.108) | (0.141) | (0.124) | (0.143) |
| Urban | 0.017 | -0.066 | 0.025 | -0.036 | -0.042 | 0.032 |
|  | (0.048) | (0.056) | (0.030) | (0.035) | (0.047) | (0.057) |
| **Adolescence factors and experiences (Age 15, Round 5)** |  |  |  |  |  |  |
| Parent-child relationship | 0.034 | -0.021 | -0.002 | -0.021 | -0.069*** | -0.059** |
|  | (0.028) | (0.026) | (0.016) | (0.018) | (0.025) | (0.025) |
| Pride Index (z-score) | -0.019 | -0.008 | -0.018 | 0.002 | -0.005 | -0.018 |
|  | (0.024) | (0.025) | (0.015) | (0.015) | (0.027) | (0.027) |
| Math Test (% Correct) | -0.033 | 0.006 | 0.005 | -0.006 | 0.091*** | 0.030 |
|  | (0.027) | (0.030) | (0.018) | (0.020) | (0.029) | (0.032) |
| Study Hours (Last Week) | -0.000 | 0.001 | -0.000 | 0.001 | 0.001 | 0.001 |
|  | (0.001) | (0.001) | (0.001) | (0.001) | (0.001) | (0.001) |
| Enrolled in School | -0.004 | -0.001 | 0.001 | 0.000 | -0.002 | 0.003 |
|  | (0.010) | (0.011) | (0.006) | (0.008) | (0.007) | (0.009) |
| BMI (z-score) | 0.023 | 0.043 | -0.012 | 0.052 | 0.187** | -0.106 |
|  | (0.082) | (0.093) | (0.077) | (0.088) | (0.086) | (0.128) |
| Change in BMI (age 5-15) | -0.005 | 0.004 | 0.002 | 0.004 | -0.008 | 0.003 |
|  | (0.008) | (0.011) | (0.007) | (0.008) | (0.008) | (0.009) |
| **Early adulthood experiences (Age 22, Round 7)** |  |  |  |  |  |  |
| High interpersonal trust | -0.015 | -0.082* | 0.002 | -0.020 | 0.031 | 0.022 |
|  | (0.037) | (0.045) | (0.021) | (0.029) | (0.053) | (0.068) |
| Big 5 Neuroticism (z-score) | 0.110*** | 0.146*** | 0.100*** | 0.063*** | 0.164*** | 0.252*** |
|  | (0.029) | (0.031) | (0.023) | (0.023) | (0.030) | (0.029) |
| Grit Index (z-score) | 0.040 | -0.069* | -0.042* | -0.031 | -0.104*** | -0.143*** |
|  | (0.032) | (0.040) | (0.025) | (0.025) | (0.035) | (0.042) |
| Food insecure | 0.070** | 0.050 | -0.058** | 0.032 | 0.072** | 0.095*** |
|  | (0.029) | (0.031) | (0.030) | (0.025) | (0.030) | (0.034) |
| Increase in the price of food I buy | 0.041 | 0.054 | 0.009 | 0.042* | 0.030 | -0.007 |
|  | (0.026) | (0.035) | (0.021) | (0.022) | (0.044) | (0.060) |
| Job loss/source of income/family enterprise | 0.095*** | 0.011 | 0.001 | 0.066 | 0.059** | 0.031 |
|  | (0.035) | (0.048) | (0.040) | (0.056) | (0.029) | (0.032) |
| Theft or destruction of property | 0.090** | 0.091** | -0.038 | 0.042 | 0.028 | -0.014 |
|  | (0.043) | (0.045) | (0.041) | (0.072) | (0.038) | (0.044) |
| Crops failed | 0.050 | 0.126* | 0.039* | 0.016 | 0.123** | 0.038 |
|  | (0.055) | (0.074) | (0.022) | (0.029) | (0.059) | (0.058) |
| Illness of a family member | 0.040 | 0.075** | 0.028 | 0.041 | 0.030 | 0.043 |
|  | (0.033) | (0.034) | (0.020) | (0.027) | (0.030) | (0.033) |
| Hours spent on caring/domestic chores past week | 0.004 | -0.004 | 0.015* | -0.002 | -0.004 | 0.001 |
|  | (0.007) | (0.006) | (0.008) | (0.005) | (0.008) | (0.006) |
| Spent 8-9 hours sleeping past week | 0.008 | -0.053* | -0.017 | -0.038 | 0.012 | -0.029 |
|  | (0.030) | (0.032) | (0.022) | (0.026) | (0.027) | (0.032) |
| Disability affecting work / capacity take care of yourself | 0.072 | 0.027 | -0.230*** | 0.204 | 0.370*** | 0.186* |
|  | (0.095) | (0.131) | (0.078) | (0.393) | (0.083) | (0.097) |
| Ever experienced IPV | 0.071 | 0.054 | 0.006 | -0.006 | 0.112*** | 0.128*** |
|  | (0.048) | (0.050) | (0.044) | (0.037) | (0.039) | (0.037) |
| Ever been bullied over the internet | 0.091* | 0.073 | 0.023 | -0.008 | 0.061 | 0.115** |
|  | (0.049) | (0.074) | (0.044) | (0.073) | (0.073) | (0.056) |
| Physical attacked due to conflict (includes attempt) | 0.038 | 0.026 |  |  |  |  |
|  | (0.030) | (0.040) |  |  |  |  |
| Ever married or cohabitating | 0.011 | 0.044 | -0.054 | -0.002 | -0.149*** | -0.011 |
|  | (0.068) | (0.056) | (0.052) | (0.036) | (0.033) | (0.043) |
| Underage marriage/cohabitation or teen parenthood | -0.200** | -0.092 | 0.117 | 0.052* | 0.059 | -0.052 |
|  | (0.084) | (0.058) | (0.092) | (0.031) | (0.053) | (0.050) |
| Ever drunk alcohol | 0.005 | -0.005 | 0.052* | 0.184* | 0.058 | 0.037 |
|  | (0.032) | (0.038) | (0.027) | (0.108) | (0.043) | (0.039) |
| Studying only | -0.036 | -0.025 | -0.045* | -0.118* | -0.019 | 0.030 |
|  | (0.048) | (0.050) | (0.027) | (0.071) | (0.051) | (0.049) |
| Working only | -0.005 | -0.019 | -0.014 | -0.075 | -0.081** | -0.036 |
|  | (0.035) | (0.050) | (0.028) | (0.070) | (0.036) | (0.042) |
| Neither working nor studying | 0.138** | -0.009 | -0.065* | -0.097 | 0.162 | -0.084 |
|  | (0.065) | (0.062) | (0.034) | (0.072) | (0.129) | (0.070) |
| Highest education level: Primary education | -0.018 | -0.049 | 0.018 | -0.038 | 0.076 | -0.024 |
|  | (0.039) | (0.052) | (0.054) | (0.066) | (0.116) | (0.223) |
| Highest education level: Secondary education | -0.007 | -0.020 | -0.019 | -0.015 | 0.029 | 0.099 |
|  | (0.040) | (0.046) | (0.053) | (0.055) | (0.117) | (0.231) |
| Highest education level: Higher education | -0.006 | -0.082 | 0.026 | -0.015 | 0.001 | 0.120 |
|  | (0.078) | (0.060) | (0.055) | (0.061) | (0.121) | (0.234) |
| Change in Wealth Index (age 1-22) | 0.141 | 0.172 | 0.099 | -0.225* | 0.045 | -0.108 |
|  | (0.119) | (0.142) | (0.100) | (0.129) | (0.113) | (0.140) |
| Migrated (age 1-22) |  |  | -0.019 | -0.046* | -0.027 | 0.059 |
|  |  |  | (0.033) | (0.028) | (0.040) | (0.052) |
| Constant | -0.069 | -0.173 | -0.009 | 0.198 | -0.064 | 0.068 |
|  | (0.179) | (0.209) | (0.147) | (0.181) | (0.186) | (0.284) |
| Observations | 619 | 612 | 935 | 781 | 764 | 794 |
| R-squared | 0.264 | 0.241 | 0.132 | 0.137 | 0.248 | 0.291 |
| Round 1 Ethnicity FE | Yes | Yes | Yes | Yes | Yes | Yes |
| Round 7 Region FE | Yes | Yes | Yes | Yes | Yes | Yes |
| Notes: Results control for missing values for the mathematics score in Round 5. We also control for missing responses on reporting IPV, bullying online, physical attacks due to conflict and drinking alcohol. Robust standard errors are reported in parentheses. *** p<0.01, ** p<0.05, * p<0.1 | | | | | | |

**References- Online Appendix**

Adhvaryu, A., Fenske, J., Nyshadham, A., 2019. Early Life Circumstance and Adult Mental Health. Journal of Political Economy 127, 1516–1549. https://doi.org/10.1086/701606

Afulani, P.A., Coleman-Jensen, A., Herman, D., 2020. Food insecurity, mental health, and use of mental health services among nonelderly adults in the United States. Journal of Hunger & Environmental Nutrition 15, 29–50. https://doi.org/10.1080/19320248.2018.1537868

Aitken, Z., Simpson, J.A., Gurrin, L., Bentley, R., Kavanagh, A.M., 2018. Do material, psychosocial and behavioural factors mediate the relationship between disability acquisition and mental health? A sequential causal mediation analysis. Int J Epidemiol 47, 829–840. https://doi.org/10.1093/ije/dyx277

Alegría, M., NeMoyer, A., Falgàs Bagué, I., Wang, Y., Alvarez, K., 2018. Social Determinants of Mental Health: Where We Are and Where We Need to Go. Curr Psychiatry Rep 20, 95. https://doi.org/10.1007/s11920-018-0969-9

Amin, V., Flores, C.A., Flores-Lagunes, A., 2020. The impact of BMI on mental health: Further evidence from genetic markers. Economics & Human Biology 38, 100895. https://doi.org/10.1016/j.ehb.2020.100895

Angelini, V., Mierau, J.O., Viluma, L., 2020. Socioeconomic Conditions in Childhood and Mental Health Later in Life, in: Zimmermann, K.F. (Ed.), Handbook of Labor, Human Resources and Population Economics. Springer International Publishing, Cham, pp. 1–18. https://doi.org/10.1007/978-3-319-57365-6_186-1

Bartram, D., 2011. Economic Migration and Happiness: Comparing Immigrants’ and Natives’ Happiness Gains From Income. Soc Indic Res 103, 57–76. https://doi.org/10.1007/s11205-010-9696-2

Beusenberg, M., Orley, J.H., Health, W.H.O.D. of M., 1994. A User’s guide to the self reporting questionnaire (SRQ.

Bhugra, D., Jones, P., 2001. Migration and mental illness. Advances in Psychiatric Treatment 7, 216–222. https://doi.org/10.1192/apt.7.3.216

Brännlund, A., Strandh, M., Nilsson, K., 2017. Mental-health and educational achievement: the link between poor mental-health and upper secondary school completion and grades. Journal of Mental Health 26, 318–325. https://doi.org/10.1080/09638237.2017.1294739

Briones, K., 2017. “How Many Rooms Are There in Your House?” Constructing the Young Lives Wealth Index.

Cleland, C., Kearns, A., Tannahill, C., Ellaway, A., 2016. The impact of life events on adult physical and mental health and well-being: longitudinal analysis using the GoWell health and well-being survey. BMC Res Notes 9, 470. https://doi.org/10.1186/s13104-016-2278-x

Coates, J., Swindale, A., Bilinsky, P., 2007. Household Food Insecurity Access Scale (HFIAS) for Measurement of Food Access: Indicator Guide: Version 3: (576842013-001). https://doi.org/10.1037/e576842013-001

Coker, A.L., Davis, K.E., Arias, I., Desai, S., Sanderson, M., Brandt, H.M., Smith, P.H., 2002. Physical and mental health effects of intimate partner violence for men and women. American Journal of Preventive Medicine 23, 260–268. https://doi.org/10.1016/S0749-3797(02)00514-7

Cunha, M., Matos, M., Faria, D., Zagalo, S., 2012. Shame Memories and Psychopathology in Adolescence: The Mediator Effect of Shame. International Journal of Psychology.

Duckworth, A.L., Peterson, C., Matthews, M.D., Kelly, D.R., 2007. Grit: Perseverance and passion for long-term goals. Journal of Personality and Social Psychology 92, 1087–1101. https://doi.org/10.1037/0022-3514.92.6.1087

Elovainio, M., Vahtera, J., Pentti, J., Hakulinen, C., Pulkki-Råback, L., Lipsanen, J., Virtanen, M., Keltikangas-Järvinen, L., Kivimäki, M., Kähönen, M., Viikari, J., Lehtimäki, T., Raitakari, O., 2020. The Contribution of Neighborhood Socioeconomic Disadvantage to Depressive Symptoms Over the Course of Adult Life: A 32-Year Prospective Cohort Study. American Journal of Epidemiology 189, 679–689. https://doi.org/10.1093/aje/kwaa026

Escobar, D.F.S.S., Noll, P.R.E.S., Jesus, T.F.D., Noll, M., 2020. Assessing the Mental Health of Brazilian Students Involved in Risky Behaviors. IJERPH 17, 3647. https://doi.org/10.3390/ijerph17103647

Fang, D., Thomsen, M.R., Nayga, R.M., 2021. The association between food insecurity and mental health during the COVID-19 pandemic. BMC Public Health 21, 607. https://doi.org/10.1186/s12889-021-10631-0

Ford, K., Freund, R., 2022. Young Lives Under Pressure: Protecting and Promoting Young People’s Mental Health at a Time of Global Crises.

Frank, P., Jokela, M., Batty, G.D., Lassale, C., Steptoe, A., Kivimäki, M., 2022. Overweight, obesity, and individual symptoms of depression: A multicohort study with replication in UK Biobank. Brain, Behavior, and Immunity 105, 192–200. https://doi.org/10.1016/j.bbi.2022.07.009

Freund, R., 2023. From drought to distress: unpacking the mental health effects of water scarcity.

Freund, R., Favara, M., Porter, C., Scott, D., Thuc Duc, L., 2022. The Mental Cost of Job Loss: Assessing the Impact on Young Adults in Vietnam. SSRN Journal. https://doi.org/10.2139/ssrn.4206223

Fryers, T., Brugha, T., 2013. Childhood Determinants of Adult Psychiatric Disorder. CPEMH 9, 1–50. https://doi.org/10.2174/1745017901309010001

Golding, J.M., 1999. Intimate Partner Violence as a Risk Factor for Mental Disorders: A Meta-Analysis. Journal of Family Violence 14, 99–132. https://doi.org/10.1023/A:1022079418229

Gruber, J., Oveis, C., Keltner, D., Johnson, S.L., 2011. A discrete emotions approach to positive emotion disturbance in depression. Cogn Emot 25, 40–52. https://doi.org/10.1080/02699931003615984

Haight, S.C., Shartle, K., Kachoria, A.G., Hagaman, A., Gupta, S., Escobar Carias, M.S., Bibi, A., Bates, L.M., Maselko, J., 2025. Female agency and probable depression in the perinatal period and beyond: Longitudinal findings from rural Pakistan. Social Science & Medicine 367, 117704. https://doi.org/10.1016/j.socscimed.2025.117704

Herba, C.M., Glover, V., Ramchandani, P.G., Rondon, M.B., 2016. Maternal depression and mental health in early childhood: an examination of underlying mechanisms in low-income and middle-income countries. Lancet Psychiatry 3, 983–992. https://doi.org/10.1016/S2215-0366(16)30148-1

Hitlin, S., Erickson, L.D., Brown, J.S., 2015. Agency and Mental Health: A Transition to Adulthood Paradox. Society and Mental Health 5, 163–181. https://doi.org/10.1177/2156869315573632

Honey, A., Emerson, E., Llewellyn, G., 2011. The mental health of young people with disabilities: impact of social conditions. Soc Psychiat Epidemiol 46, 1–10. https://doi.org/10.1007/s00127-009-0161-y

Howard, L.M., MacManus, D., 2020. Domestic violence and abuse and mental health, in: Geddes, J.R., Andreasen, N.C., Goodwin, G.M., Geddes, J.R., Andreasen, N.C., Goodwin, G.M. (Eds.), New Oxford Textbook of Psychiatry. Oxford University Press, p. 0. https://doi.org/10.1093/med/9780198713005.003.0143

Jao, N.C., Robinson, L.D., Kelly, P.J., Ciecierski, C.C., Hitsman, B., 2019. Unhealthy behavior clustering and mental health status in United States college students. Journal of American College Health 67, 790–800. https://doi.org/10.1080/07448481.2018.1515744

John, O.P., Srivastava, S., 1999. The Big Five Trait taxonomy: History, measurement, and theoretical perspectives, in: Handbook of Personality: Theory and Research, 2nd Ed. Guilford Press, New York, NY, US, pp. 102–138.

Knight, J., Gunatilaka, R., 2010. Great Expectations? The Subjective Well-being of Rural–Urban Migrants in China. World Development 38, 113–124. https://doi.org/10.1016/j.worlddev.2009.03.002

Koenen, K.C., Moffitt, T.E., Roberts, A.L., Martin, L.T., Kubzansky, L., Harrington, H., Poulton, R., Caspi, A., 2009. Childhood IQ and Adult Mental Disorders: A Test of the Cognitive Reserve Hypothesis. AJP 166, 50–57. https://doi.org/10.1176/appi.ajp.2008.08030343

Kruger, D.I., 2004. Coffee Production Effects on Child Labor and Schooling in Rural Brazil. https://doi.org/10.2139/ssrn.625345

Kurniyawan, E.H., Septia Pratiwi, D., Kurniasari, A., Putri Sonya, K., Khoiro Maulidia, I., Rosyidi Muhammad Nur, K., Endrian Kurniawan, D., 2024. Management Stress On Farmers In Agricultural Areas. NHSJ 4, 96–102. https://doi.org/10.53713/nhsj.v4i1.333

Landstedt, E., Coffey, J., Wyn, J., Cuervo, H., Woodman, D., 2017. The Complex Relationship between Mental Health and Social Conditions in the Lives of Young Australians Mixing Work and Study. YOUNG 25, 339–358. https://doi.org/10.1177/1103308816649486

Leight, J., Pedehombga, A., Ganaba, R., Gelli, A., 2022. Women’s empowerment, maternal depression, and stress: Evidence from rural Burkina Faso. SSM - Mental Health 2, 100160. https://doi.org/10.1016/j.ssmmh.2022.100160

Marsh, H.W., O’Neill, R., 1984. Self Description Questionnaire III: The Construct Validity of Multidimensional Self-Concept Ratings by Late Adolescents. Journal of Educational Measurement 21, 153–174.

Martínez, L.M., Estrada, D., Prada, S.I., 2019. Mental health, interpersonal trust and subjective well-being in a high violence context. SSM - Population Health 8, 100423. https://doi.org/10.1016/j.ssmph.2019.100423

Meekers, D., Pallin, S.C., Hutchinson, P., 2013. Intimate partner violence and mental health in Bolivia. BMC Women’s Health 13, 28. https://doi.org/10.1186/1472-6874-13-28

Melchior, M., Moffitt, T.E., Milne, B.J., Poulton, R., Caspi, A., 2007. Why do children from socioeconomically disadvantaged families suffer from poor health when they reach adulthood? A life-course study. Am J Epidemiol 166, 966–974. https://doi.org/10.1093/aje/kwm155

Musumari, P.M., Tangmunkongvorakul, A., Srithanaviboonchai, K., Techasrivichien, T., Suguimoto, S.P., Ono-Kihara, M., Kihara, M., 2018. Grit is associated with lower level of depression and anxiety among university students in Chiang Mai, Thailand: A cross-sectional study. PLoS ONE 13, e0209121. https://doi.org/10.1371/journal.pone.0209121

Nebhinani, N., Jain, S., 2019. Adolescent Mental Health: Issues, Challenges, and Solutions. Annals of Indian Psychiatry 3, 4. https://doi.org/10.4103/aip.aip_24_19

Nesbitt, A.E., Pila, E., Crocker, P.R.E., Sabiston, C.M., 2023. Global and body-related self-conscious emotions: exploring associations to positive and negative mental health. Self and Identity 22, 809–831. https://doi.org/10.1080/15298868.2023.2184858

Newton-Howes, G., Horwood, J., Mulder, R., 2015. Personality characteristics in childhood and outcomes in adulthood: Findings from a 30 year longitudinal study. Aust N Z J Psychiatry 49, 377–386. https://doi.org/10.1177/0004867415569796

Njeru, M.W., Arasa, J.N., Musau, J.N., Kihara, M., 2022. The Effects of Climate Change on the Mental Health of Smallholder Crop Farmers in Embu and Meru Counties of Kenya. Afr. j. clim. chang. resour. sustain. 1, 1–12. https://doi.org/10.37284/ajccrs.1.1.667

Oh, H., Goehring, J., Jacob, L., Smith, L., 2021. The Environment of Birthplace and Self-Reported Mental Health Conditions: Findings from the American Panel of Life. Epidemiologia 2, 256–261. https://doi.org/10.3390/epidemiologia2030019

Orth, U., Robins, R.W., Soto, C.J., 2010. Tracking the trajectory of shame, guilt, and pride across the life span. J Pers Soc Psychol 99, 1061–1071. https://doi.org/10.1037/a0021342

Patel, V., Kirkwood, B.R., Pednekar, S., Pereira, B., Barros, P., Fernandes, J., Datta, J., Pai, R., Weiss, H., Mabey, D., 2006. Gender disadvantage and reproductive health risk factors for common mental disorders in women: a community survey in India. Arch Gen Psychiatry 63, 404–413. https://doi.org/10.1001/archpsyc.63.4.404

Patel, V., Kleinman, A., 2003. Poverty and common mental disorders in developing countries. Bulletin of the World Health Organization.

Peterson, L.E., Tsai, A.C., Petterson, S., Litaker, D.G., 2009. Rural-urban comparison of contextual associations with self-reported mental health status. Health Place 15, 125–132. https://doi.org/10.1016/j.healthplace.2008.03.001

Porter, C., Favara, M., Hittmeyer, A., Scott, D., Sánchez Jiménez, A., Ellanki, R., Woldehanna, T., Duc, L.T., Craske, M.G., Stein, A., 2021. Impact of the COVID-19 pandemic on anxiety and depression symptoms of young people in the global south: evidence from a four-country cohort study. BMJ Open 11, e049653. https://doi.org/10.1136/bmjopen-2021-049653

Revollo, P.E., Scott, D., 2022. Cognitive and achievement tests in the Young Lives Study.

Roelofs, J., Huibers, M., Peeters, F., Arntz, A., Van Os, J., 2008. Rumination and worrying as possible mediators in the relation between neuroticism and symptoms of depression and anxiety in clinically depressed individuals. Behaviour Research and Therapy 46, 1283–1289. https://doi.org/10.1016/j.brat.2008.10.002

Rosenberg, M., 1965. Society and the Adolescent Self-Image. JSTOR.

Sharma, P., Uzar, K., Brelsford, G.M., 2021. Self-Esteem, Grit, and Optimism: Predictors of Depression and Anxiety in Emerging Adults. Journal of College Student Development 62, 563–574.

Shen, W., Hannum, E., 2023. Context-relevant risk and protective factors for children in rural communities: Long-term implications for adulthood educational and mental health outcomes. J Community Psychol 51, 724–744. https://doi.org/10.1002/jcop.22909

Smith, D.T., Mouzon, D.M., Elliott, M., 2018. Reviewing the Assumptions About Men’s Mental Health: An Exploration of the Gender Binary. Am J Mens Health 12, 78–89. https://doi.org/10.1177/1557988316630953

Stansfeld, S., Candy, B., 2006. Psychosocial work environment and mental health—a meta-analytic review. Scand J Work Environ Health 32, 443–462. https://doi.org/10.5271/sjweh.1050

Steptoe, A., Frank, P., 2023. Obesity and psychological distress. Philos Trans R Soc Lond B Biol Sci 378, 20220225. https://doi.org/10.1098/rstb.2022.0225

Stillman, S., Gibson, J., McKenzie, D., Rohorua, H., 2015. Miserable Migrants? Natural Experiment Evidence on International Migration and Objective and Subjective Well-Being. World Development, Migration and Development 65, 79–93. https://doi.org/10.1016/j.worlddev.2013.07.003

Van Doren, N., Tharp, J.A., Johnson, S.L., Staudenmaier, P.J., Anderson, C., Freeman, M.A., 2019. Perseverance of effort is related to lower depressive symptoms via authentic pride and perceived power. Personality and Individual Differences 137, 45–49. https://doi.org/10.1016/j.paid.2018.07.044

Veldman, K., Bültmann, U., Stewart, R.E., Ormel, J., Verhulst, F.C., Reijneveld, S.A., 2014. Mental Health Problems and Educational Attainment in Adolescence: 9-Year Follow-Up of the TRAILS Study. PLoS ONE 9, e101751. https://doi.org/10.1371/journal.pone.0101751

Ventriglio, A., Torales, J., Castaldelli-Maia, J.M., Berardis, D.D., Bhugra, D., 2021. Urbanization and emerging mental health issues. CNS Spectrums 26, 43–50. https://doi.org/10.1017/S1092852920001236

von Russdorf, S., Ahlborn, L., Hidalgo-Arestegui, A., McQuade, G., Favara, M., 2024. A sound methodology: Measuring experiences of violent conflict through audio self-interviews. Economics Letters 242, 111879. https://doi.org/10.1016/j.econlet.2024.111879

Weckström, T., Elovainio, M., Pulkki-Råback, L., Suokas, K., Komulainen, K., Mullola, S., Böckerman, P., Hakulinen, C., 2023. School achievement in adolescence and the risk of mental disorders in early adulthood: a Finnish nationwide register study. Mol Psychiatry 28, 3104–3110. https://doi.org/10.1038/s41380-023-02081-4

Yorke, L., Ogando Portela, M.J., 2018. Psychosocial Scales in the Young Lives Round 4 Survey: Selection, Adaptation and Validation.

Yount, K.M., Dijkerman, S., Zureick-Brown, S., VanderEnde, K.E., 2014. Women’s empowerment and generalized anxiety in Minya, Egypt. Social Science & Medicine 106, 185–193. https://doi.org/10.1016/j.socscimed.2014.01.022
